# The inside out model of emotion recognition: how the shape of one’s internal emotional landscape influences the recognition of others’ emotions

**DOI:** 10.1038/s41598-023-48469-8

**Published:** 2023-12-06

**Authors:** Connor Tom Keating, Jennifer Louise Cook

**Affiliations:** University of Birmingham, Birmingham, UK

**Keywords:** Human behaviour, Psychology

## Abstract

Some people are exceptional at reading emotional expressions, while others struggle. Here we ask whether the way we experience emotion “on the inside” influences the way we expect emotions to be expressed in the “outside world” and subsequently our ability to read others’ emotional expressions. Across multiple experiments, incorporating discovery and replication samples, we develop EmoMap (N = 20; N = 271) and ExpressionMap (N = 98; replication N = 193) to map adults’ experiences of emotions and visual representations of others’ emotions. Some individuals have modular maps, wherein emotional experiences and visual representations are consistent and distinct—anger looks and feels different from happiness, which looks and feels different from sadness. In contrast, others have experiences and representations that are variable and overlapping—anger, happiness, and sadness look and feel similar and are easily confused for one another. Here we illustrate an association between these maps: those with consistent and distinct *experiences* of emotion also have consistent and distinct *visual representations* of emotion. Finally (N = 193), we construct the *Inside Out Model of Emotion Recognition,* which explains 60.8% of the variance in emotion recognition and illuminates multiple pathways to emotion recognition difficulties. These findings have important implications for understanding the emotion recognition difficulties documented in numerous clinical populations.

## Introduction

Some people are exceptional at navigating the social world: the considerate concierge rapidly reads facial expressions and anticipates every desire; the perceptive companion accurately detects the sadness behind their friend’s smile; the skilled negotiator notices a telling tightness around the eyes and knows just the right time to apply pressure. Other individuals struggle: as Parkinson’s Disease progresses, people with this condition increasingly report challenges with reading others’ emotional expressions^1^, and similar difficulties predict negative social and wider health outcomes across a range of psychiatric and mental health conditions^2–4^. Despite clear individual differences in the ability to read others’ emotional expressions, little is known about why these individual differences exist. Here we ask whether individual differences in navigating the social world of others’ facial expressions are related to individual differences in the shape of one’s own *internal* emotional landscape. In other words, is there a relationship between our experience of emotion “on the inside” and our ability to identify emotions in the “outside world”?

Internal “maps” of concepts—such as personality traits—can exert a considerable influence on the judgments we make about others. Stolier et al.^5^ for instance, mapped internal conceptual-trait maps by asking participants to rate the similarity of 13 different personality traits. They also mapped representations of how these traits are depicted on people’s faces by asking participants to rate various face images with respect to these 13 traits. Both internal (semantic) conceptual maps and external maps of facial representations, tended to exhibit a modular structure with particular traits—such as aggressive, mean, dominant and egotistical—clustering together. Importantly, the shape of an individual’s map of facial representations was highly correlated with the shape of their internal conceptual landscape, such that a perceiver who believed aggression and dominance to be closely related in conceptual space would be more likely (compared to a perceiver with a weak link between the two concepts) to see an aggressive face as dominant. Thus, Stolier and colleagues illustrate that, for trait judgements, internal conceptual maps and judgements we make about others in the outside world are tightly related.

Stolier and colleagues' work pertains to traits. Here we focus on emotions. Preliminary evidence provides initial support for a link between the experience and recognition of emotion. Israelashvili and colleagues^6^ for example, illustrated that individuals who are good at differentiating their own experiences of distinct emotions are more accurate in reading others' emotional facial expressions. Nevertheless, although preliminary evidence indicates that individuals who are better able to identify how they feel “on the inside” are also better able to recognize emotions in the “outside world”, it is unclear why this relationship exists. Afterall, recognizing *one’s own* emotions primarily depends upon the labelling of *internal signals*, whereas recognizing *others’* emotions principally consists of categorizing *incoming sensory information*. The psychological mechanisms supporting superior emotion recognition in individuals with superior (own) emotion differentiation are currently unknown.

The face identity literature provides a candidate mechanism: studies from this field have illustrated that individuals who are good at face identity recognition tend to have robust visual representations (also referred to as templates and/or abstracted structural representations) of others’ identities, in their minds eye^7–9^. Such representations are thought to be constructed via experience wherein exposure to different views of a face updates the abstracted structural representation of this identity and, over time, the representation comes to emphasize diagnostic aspects of the face (that differentiate this face from another) and minimize non-diagnostic aspects. Signal detection theory (see^10^) also tells us that distinguishing between signal and noise (e.g., correct and incorrect facial identities) is easier if the signal and noise distributions are distinct and precise—when these channels are not overlapping, and when they are *consistent* across numerous instances or samples (i.e., narrow). In line with this*,* Etchells et al.^9^ found that participants were better at recognizing faces from a novel view when they had built up a more precise representation of that face from multiple views, relative to a single view, during a preceding learning phase. Furthermore, it is well documented that faces that are more overlapping in appearance are more difficult to differentiate^11^. Therefore, the face identity literature raises the hypothesis that individuals who are adept at reading others’ emotions will have *consistent* and *distinct* visual representations (in their ‘mind’s eye’) of emotional facial expressions. This hypothesis is yet to be tested.

If we are to understand why people who are better at recognizing *others’* emotions tend to be good at identifying *their own*, and if this is related to the consistency of visual representations of *others’* emotional expressions, we must also explain why representations would be more consistent and distinct for individuals who are better able to differentiate their *own* emotions. Models of conceptual learning suggest that robust concepts facilitate learning: having a (semantic) concept that a table has a flat top and four legs encourages a learner to focus on these invariant features when encountering new table exemplars and ignore variant features such as color or texture^12,13^, thus minimizing within-category differences and maximizing between-category differences^14^. Similarly, having consistent and distinct concepts of *one’s own* emotions (which may be multidimensional including semantic, interoceptive and sensory information^15–20^) may encourage a learner to focus on invariant features of facial expressions and ignore between expression variation, thus encouraging the formation of consistent and distinct visual representations of *others’* facial expressions. However, despite theoretical justification for a link between the experience and representation of emotion, research has not yet tested this idea.

Here we ask whether the experience of emotion “on the inside” influences the way in which one represents the dynamic emotional facial expressions that one would encounter in the “outside world” and whether this, in turn, affects emotion recognition accuracy. Specifically, we predict that some individuals will have internal emotion maps with a clear modular structure, wherein emotional experiences are *consistent* and *distinct*: happiness feels very different from anger, which feels very different from sadness. We predict that these individuals will also have *consistent* and *distinct* representations of the way in which emotions are expressed on others’ faces and, correspondingly, will be adept at recognizing expressions. Other individuals, however, may have *variable* and *overlapping* experiences of emotion wherein anger, happiness and sadness feel relatively similar and are easily confused for one another. We predict that these individuals will have more *variable* and *overlapping* visual representations of others’ expressions such that, in their mind’s eye, anger, happiness and sadness look relatively similar. Thus, resulting in emotion recognition difficulties.

Across a series of experiments, we first develop and validate “EmoMap”, a novel method to map the shape of individuals’ emotional experience landscapes (Expt 1). Second, we develop “ExpressionMap” to map the landscape of participants’ visual representations of emotional expressions (Expt 2). Following this, we test for a mapping between the experience of emotion “on the inside” and representations of the way emotions are expressed in the “outside world”. That is, we ask whether those with modular internal emotion maps, who have consistent and distinct experiences of anger, happiness and sadness, also tend to have consistent and distinct visual representations of angry, happy and sad facial expressions (note that these emotions were selected as they correspond to different regions in the circumplex model of emotion^21^, varying in both and valence). Throughout these analyses, we control for clinically relevant demographic factors known to be associated with the experience and perception of emotion (e.g., the level of autistic traits, the level of alexithymic traits, and non-verbal reasoning ability^22–28^) to ensure that any relationships we discover exist even after accounting for these variables. Finally, we assess the contribution of the consistency and differentiation (i.e., distinctness) of emotional experiences and representations to the recognition of anger, happiness and sadness, and use structural equation modelling to construct the ‘Inside Out Model’ of emotion recognition; a model which provides insight into the psychological mechanisms by which one’s experience of emotions “on the inside” influences one’s ability to identify emotions in the “outside world”.

## Results

### Study 1: developing EmoMap

Participants (*N* = 271) completed our EmoMap paradigm—a two-part task that assesses the differentiation and consistency of emotional experiences. In the first part, on each trial, participants viewed pairs of images (from the Nencki Affective Picture System^29^) each known to selectively induce either anger, happiness or sadness^30^, and were asked to rate how similar the emotions evoked by the images were on a scale from zero, ‘Not at all similar’, to ten, ‘Very similar’ (to 4 decimal places). These similarity scores were then transformed into distance scores via multidimensional scaling, a statistical technique that represents objects (emotional images, lexical items) as points in multidimensional space, wherein close similarity between objects corresponds to small distances between the points in the representation. Distance scores were then used to (a) calculate the mean distances between (e.g., distance between angry and happy clusters, angry and sad clusters, and happy and sad clusters) and within emotion clusters, and (b) plot multidimensional scaling maps.

**Figure 1:**
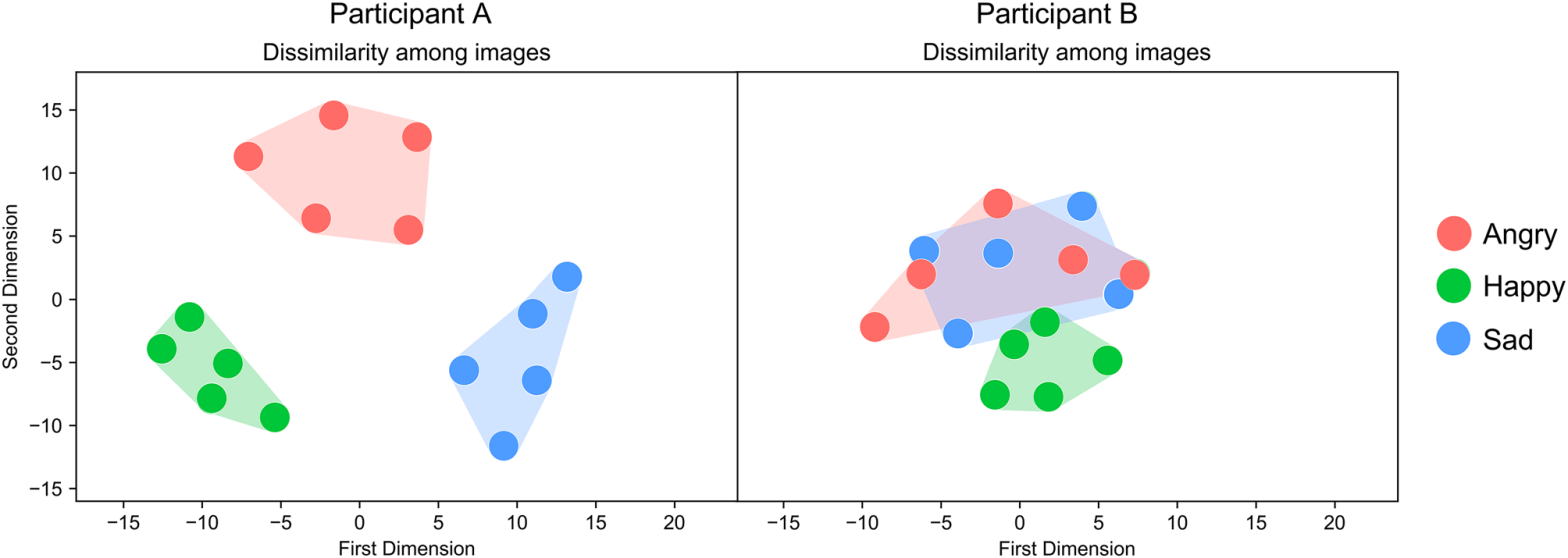
Examples of consistent and distinct (left), and variable and overlapping (right) emotional landscapes. The dimensions illustrated here may somewhat reflect the two dimensions outlined in the circumplex model of affect^21^—arousal and valence. The first dimension may correspond to valence, with high values reflecting negative valence and low values reflecting positive valence (see left). The second dimension may correspond to arousal; high scores reflect high activation, and low scores reflect low activation (see left). This may be an appropriate interpretation of the internal emotional landscape for Participant A (left).

The multidimensional scaling maps confirmed that the internal emotional landscape had a modular structure for some participants (Fig. 1, left panel) and a less modular, more overlapping, structure for others (Fig. 1, right panel)*.* EmoMap was validated by illustrating that individuals high in alexithymic traits, who by definition have difficulties differentiating their own emotions^31^, tended to have emotional landscape maps with a less modular, more overlapping structure, whereas those low in alexithymic traits had modular emotional landscapes. That is, linear mixed effects models predicting mean distance between clusters and mean distance within clusters with TAS score, AQ score, and non-verbal reasoning ability (clinically relevant demographic variables known to be associated with the experience and perception of emotion; e.g.,^22–28^), and with subject number as a random intercept revealed alexithymic traits as a significant negative predictor of distance *between* emotion clusters [F(1,267) = − 5.92, *p* < 0.05] and distance *within* emotion clusters [F(1,267) = − 6.16, *p* < 0.05]. In general, greater overlap was seen between anger and sadness [mean distance (SEM) = 14.39 (0.21)], than happiness and anger [mean distance (SEM) = 20.79 (0.29)], and happiness and sadness [mean distance (SEM) = 20.70 (0.29)] in participants’ internal emotional landscapes (see Supplementary Information A for a full discussion). These results validate EmoMap by confirming that individuals who, by definition, have difficulties differentiating their own emotions exhibit higher EmoMap emotion confusion as indexed by smaller distances between- and within- emotion clusters (suggesting they have difficulties differentiating distinct and more similar emotional states).

In the second part of EmoMap, on each trial participants were required to make decisions about three images (also from the Nencki Affective Picture System^29^). There were four conditions: one non-emotional control condition, and three emotional experimental conditions exploring the experience of anger, happiness and sadness respectively. Participants completed the control condition first. In this condition, participants were required to select which of the three (emotionally neutral) images they found most colorful using their mouse cursor. Two of these images were in color and one was in grayscale, thus serving as an attention check. Following this, participants completed the three experimental conditions in a random order. In these conditions, participants were required to select which of the three images made them feel most angry, happy or sad using their mouse cursor (i.e., in the ‘angry condition’ participants would have to decide which image made them most angry). As in the control condition, there was a ‘trap’ image on each trial such that two of the images were strong inducers of the target emotion (e.g., sadness), and one was a strong inducer of another emotion (e.g., happiness), thus serving as an attention check. Emotional consistency was calculated, for each emotion, based on the logical consistency of decision-making: if a participant selected image A over image B (A > B) and image B over image C (B > C), but then selected image C over image A (C > A), this would be considered an inconsistent decision and would result in a reduction in their consistency score (see Methods for further details on scoring). Consistency requires participants to differentiate precisely between the intensity of emotion evoked by each image. Therefore, inconsistent decisions are likely to stem from inconsistencies in an individual’s emotional experience across repeated instances.

Using scores from this task, we aimed to determine whether there is a link between the consistency and differentiation of emotional experiences. Our results illustrate that individuals with modular landscapes are more likely to have consistent emotional experiences, whereas those with more overlapping emotional landscapes have less reliable emotional experiences. That is, a linear mixed effects model of emotional consistency as a function of between-cluster distances, within-cluster distances, the interaction between emotion and between-cluster distances, the interaction between emotion and within-cluster distances (independent variables), AQ, TAS, non-verbal reasoning and color (control) consistency (control variables), with subject number as a random intercept revealed that emotional consistency was positively predicted by between-cluster distances [F(1,786.1) = 9.58, p < 0.01], and negatively predicted by within-cluster distances [F(1,785.9) = − 10.30, p < 0.01]. Since the emotion that was displayed (angry, happy or sad) did not interact with between- or within-cluster distances to predict emotional consistency, our results suggest that those with larger distances *between* clusters and smaller distances *within* their emotion clusters typically had greater emotional consistency for anger, happiness *and* sadness. Emotional consistency was also positively predicted by non-verbal reasoning ability [F(1,264) = 12.83, *p* < 0.001] but not by any other variables, including color control consistency [all *p* > 0.05]. Hence, our results demonstrate that the distances between and within emotion clusters predict the consistency of emotional experiences.

### Study 2: developing ExpressionMap

To map visual representations of the external expression landscape, participants (*N* = 98; replication *N* = 193) completed our ExpressionMap paradigm. On each trial participants were asked to move a dial to change the speed of an emotional point light display of the face (a PLF) until it matched the speed they typically associated with an angry, happy or sad expression. That is, participants were matching the speed of the displayed PLF to their visual representation of that expression. The consistency of visual representations was indexed as the standard deviation of the speeds attributed to each repetition of the angry, happy and sad expressions respectively, multiplied by -1 (see “Methods” for full details). Mean representational consistency was calculated by taking a mean of the consistency scores for the angry, happy and sad PLFs. In addition, this task also provides an index of the ‘distance’ between emotions in participants’ visual representations of facial expressions. Distance scores were calculated as the absolute difference in the speeds attributed to two different emotions. For example, to calculate distance between happy and angry, we subtracted the mean speed attributed to happy PLFs from the mean speed attributed to angry PLFs, and then took the absolute value. Mean distance was calculating by taking a mean of the scores for the angry-happy, angry-sad, and happy-sad distances.

**Figure 2:**
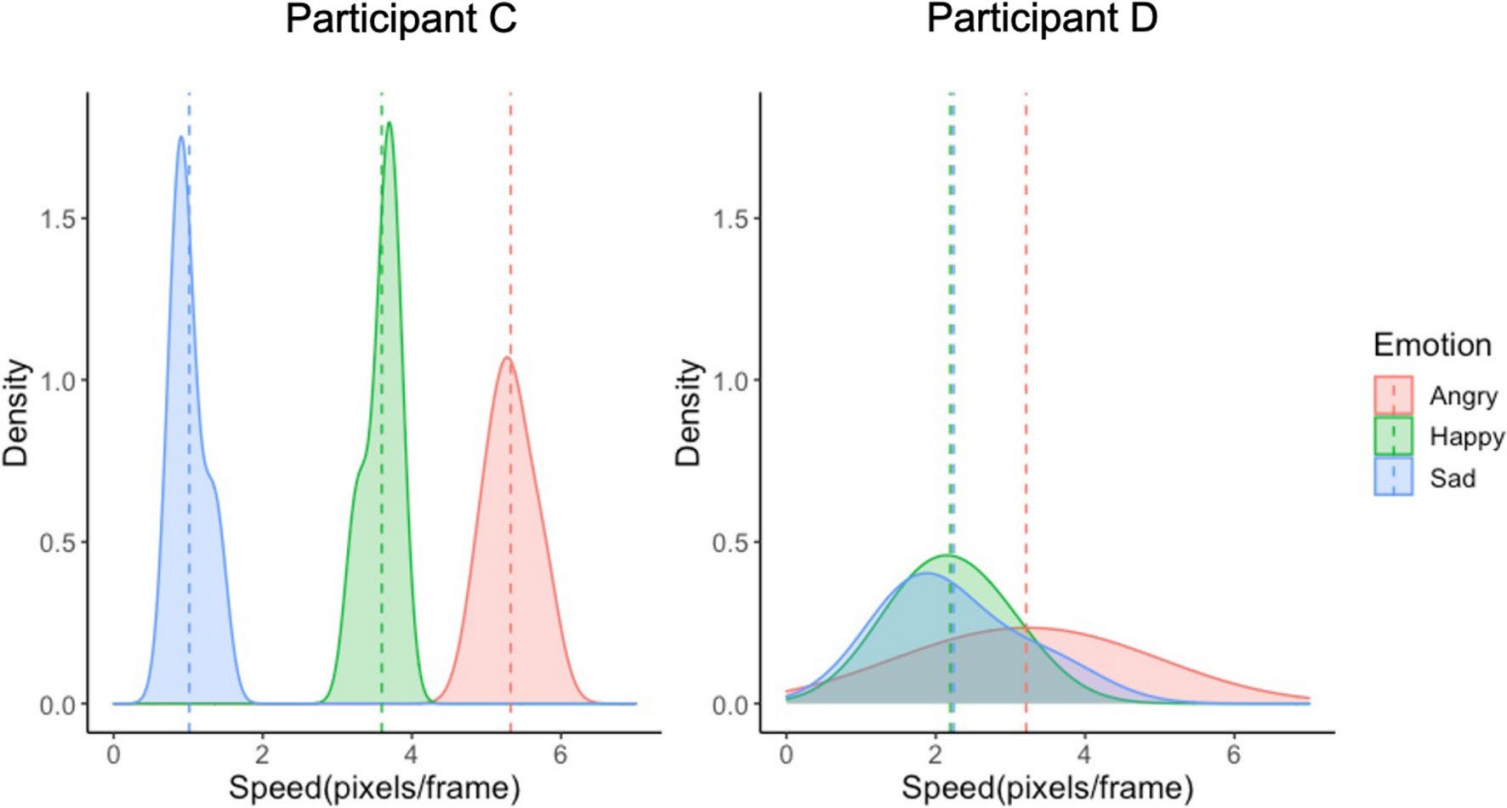
Examples of consistent and distinct (left), and variable and overlapping (right) visual emotion representations.

To visualize representations of the external emotional landscape, we produced density plots displaying the speeds attributed to angry, happy and sad expressions respectively. Density plots confirmed that for some individuals, visual representations of emotion are *consistent* and *distinct* (Fig. 2, left panel), and for others they are *variable* and *overlapping* (Fig. 2, right panel). Across participants, the consistency and differentiation of such representations differed as a function of emotion/emotion pair—these results are reported in Supplementary Information A as they are outside the scope of the current study.

#### Mapping between the experience of emotion “on the inside” and representations of emotional expressions in the “outside world”

**Figure 3:**
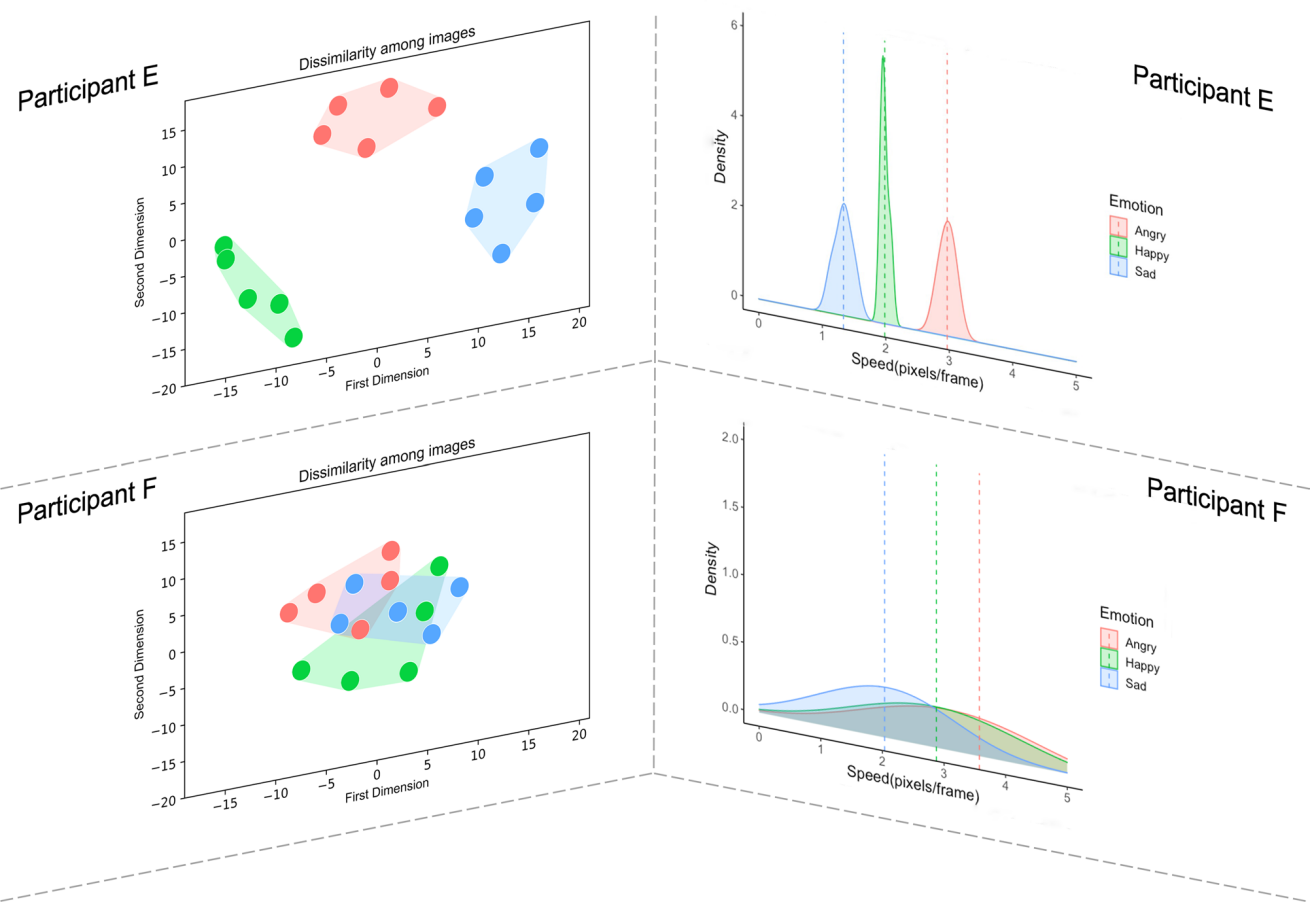
A diagram demonstrating that consistency and differentiation within internal emotional landscapes (left) is linked to consistency and differentiation in visual models of the external world (right). Figure top shows the modular emotion and representation maps of one participant. Figure bottom shows the overlapping emotion and representation maps of another participant.

A subset of participants (N = 193) completed both EmoMap and ExpressionMap. To probe the existence of a mapping between the experience of emotion “on the inside” and representations of the way emotions are expressed in the “outside world”, we constructed two separate linear mixed effects models to predict metrics of ExpressionMap (representational consistency and distance between representations) from metrics derived from EmoMap (emotional consistency, distance between emotion clusters, respectively), with AQ score, TAS score, non-verbal reasoning, and color (control) consistency as control variables, and with subject number as a random intercept. Representational consistency was positively predicted by emotional consistency [F(1,186) = 5.15, *p* < 0.05] but not colour control consistency [p > 0.05]: individuals with more consistent experiences of emotion also had more consistent visual representations of emotion (while the consistency of colorfulness judgments did not contribute to the consistency of visual representations). Non-verbal reasoning was also a significant predictor of representational consistency [F(1,186) = 30.71, p < 0.001]: those with higher non-verbal reasoning had greater representational consistency. In addition, distance between emotion representations was predicted by distance between emotion clusters [F(1,186) = 8.19, *p* < 0.01]: those with more distinct experiences of emotion also had more distinct representations. Thus, consistency and differentiation within internal emotional landscapes is linked to consistency and differentiation in visual representations of the external world (even after controlling for relevant participant demographics; see Fig. 3).

#### Predicting emotion recognition ability

The above analyses illustrate a mapping between the experience of emotion “on the inside” and visual representations of the way emotions are expressed in the “outside world”, but how do these inside and outside maps influence emotion recognition accuracy? To answer this question, we first focused on asking how the shape of ExpressionMaps relate to individual differences in emotion recognition as indexed by our previously validated PLF Emotion Recognition Task^22,32^. On each trial in this task, participants viewed an angry, happy or sad PLF and rated the extent to which the expression looked angry, happy and sad. Emotion recognition accuracy was calculated as the correct emotion rating minus the mean of the two incorrect emotion ratings.

**Figure 4:**
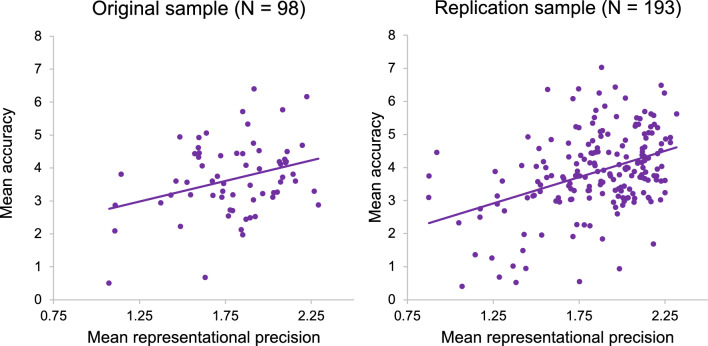
The relationship between mean accuracy and mean representational consistency in original sample (left [R = 0.311, p = 0.002, BF_10_ = 15.32]) and replication sample (right [R = 0.399, p < 0.001, BF_10_ = 1.21e^6^]).

Building on the face identity literature^7–9^ and principles of signal detection theory^10^, our a priori hypothesis was that emotion recognition accuracy would be positively predicted by the consistency of, and distance between, emotion representations. To test this, we constructed a linear mixed effects model with accuracy as the outcome variable, representational consistency, distance between emotion representations, the interaction between representational consistency and distance, AQ score, TAS score, and non-verbal reasoning as predictors (clinically relevant participant characteristics known to be involved in the experience and perception of emotion; e.g.,^22–28^), and subject number as a random intercept. Across both our original (*N* = 98) and replication (*N* = 193) sample, representational consistency was a significant positive predictor of accuracy [original sample: F(1,91) = 4.19, *p* < 0.05; replication sample: F(1,186) = 13.86, *p* < 0.001; see Fig. 4]: those with more consistent visual emotion representations typically achieved higher accuracy (i.e. identified the emotion that the actor intended to convey) on the PLF Emotion Recognition Task. In conflict with our hypothesis, accuracy was not predicted by distance in either sample [all *p* > 0.05]. There were also no other significant predictors of accuracy across both samples [all *p* > 0.05].

Since it is likely that emotion recognition is contingent not only on the clarity of emotion representations but also on the ability to match a displayed expression to one’s visualization, we also included a visual matching task in our battery. This task assesses how well participants can visually match the speed of one expression to another displayed expression. Each trial began with a PLF stimulus video on the left-hand side of the screen. After this video had played once, the same PLF stimulus video also appeared on the right-hand side of screen (moving at a random speed) and continued to play in a loop. Participants were instructed to “move the dial to change the speed of the video on the right until it matches the speed of the video on the left”. Consequently, participants were visually matching the speed of one PLF to another. Deviation scores (the distance between the speeds of the two animations) comprised the absolute value of the percentage speed attributed to the leftward expression minus that attributed to the rightward expression. Mean deviation scores comprised a mean of all of the absolute deviation scores. Higher deviation scores represented greater difficulties matching the two expressions.

**Figure 5:**
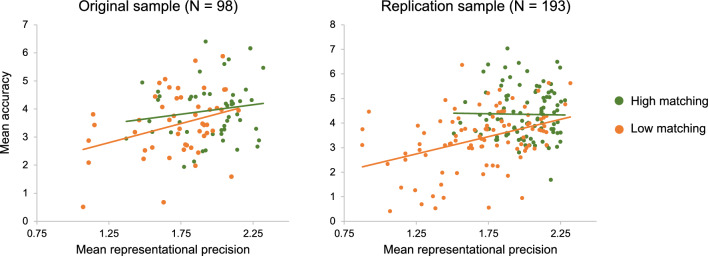
The relationship between mean accuracy and representational consistency within the high (original sample: R = 0.148, p = 0.310, BF_10_ = 0.294; replication sample: R = 0.004, p = 0.972, BF_10_ = 0.13) and low matching groups (original sample: R = 0.324, p < 0.05, BF_10_ = 2.18; replication sample: R = 0.379, p < 0.001, BF_10_ = 176.06).

Subsequently, visual matching difficulty and the interaction between representational consistency and matching difficulty were added to the linear mixed effects model described above. In our larger sample, we found that the main effect of representational consistency on emotion recognition accuracy was moderated by matching difficulty [F(1,184) = 12.26, *p* < 0.001]. To unpack this interaction, we conducted a median half split analysis, dividing participants into a high matching group (matching deviation scores < 27.75%) and a low matching group (matching deviation scores > 27.75%). Representational consistency was only a significant predictor of accuracy for those with lower matching ability [F(1,89) = 7.16, *p* < 0.01], and not those with higher matching ability [F(1,90) = 0.44, *p* = 0.507] (see Fig. 5). This interaction was also evident in our original sample [low matching: F(1,42) = 4.18, *p* < 0.05; high matching: F(1,42) = 0.44, *p* = 0.513]. Hence, across both samples, for participants with a lower ability to match expressions, representational consistency was a significant predictor of emotion recognition ability. This potentially indicates that when one’s ability to match two representations is compromised, having clear and consistent visual representations becomes particularly important.

In sum, emotion recognition ability is predicted by the consistency of imagined visual emotion representations and one’s matching ability, such that—for individuals with lower ability to match two visually displayed expressions—the more consistent one’s representations the better one’s emotion recognition accuracy.

#### Building the inside out model of emotion recognition (N = 193)

For the following analyses, we focused on the 193 participants that had completed all four tasks (EmoMap, ExpressionMap, Visual Matching and PLF Emotion Recognition), thus allowing us to build a comprehensive model incorporating the experience, representation and recognition of emotion. Model construction comprised a four-step process. First, since we had many potential variables of interest, we determined their relative importance for emotion recognition using a random forests analysis^33^ employing the Boruta wrapper algorithm^34^. In this analysis, representation matching, matching difficulty, representational consistency, distance between emotion clusters, and emotional consistency were deemed important for emotion recognition. Here, ‘representation matching’ reflects the interaction between representational consistency and matching difficulty, which was found to be a significant predictor of emotion recognition in our previous analyses. ‘Representation matching’ was computed by multiplying the representational consistency scores for angry, happy and sad expressions with their corresponding matching difficulty scores (e.g., angry representational consistency x angry matching difficulty; happy representational consistency x happy matching difficulty; sad representational consistency x sad matching difficulty). Higher representation matching scores indicate superior representational consistency, matching ability, or both. Following our random forests analysis, we added variables classified as “important” into a structural equation model predicting emotion recognition accuracy, sequentially (starting with the most important variable), until there was no longer a significant improvement (or our goodness of fit index exceeded the specified threshold). Third, to determine the most mathematically plausible path directions in our structural equation model, we systematically reversed each path and compared the Bayesian Information Criterion (BIC) scores for the original and reversed models (see Supplementary Information B for the steps listed above). Lastly, we built one final structural equation model in which we included the path directions that were mathematically most plausible. There was strong to very strong evidence that this final model was better than all previous models (BIC difference > 6).

**Figure 6:**
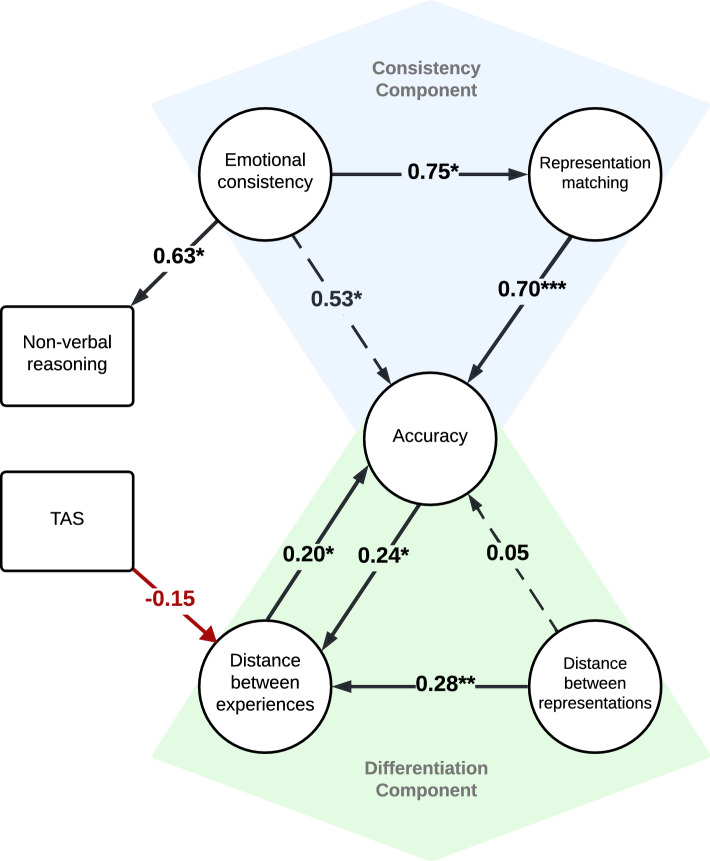
The final structural equation model exploring the experience, visualization and recognition of emotion. Circles correspond with latent variables; rectangles correspond with manifest variables. Black arrows indicate positive relationships. Red arrows indicate negative relationships. Full line arrows correspond with direct effects; dashed line arrows correspond with indirect effects. The values displayed are the standardized beta path coefficients. The significance level for direct and indirect effects are shown by asterisks: *p < 0.05, **p ≤ 0.01, ***p ≤ 0.001.

In our final structural equation model (see Fig. 6), which could account for 60.8% of the variance in emotion recognition accuracy, there were two component processes that contributed to individual differences: the *consistency component* and the *differentiation component*. With respect to the former, emotional consistency exerted an indirect effect on emotion recognition [z = 2.05, *b* = 0.53, *p* < 0.05], by influencing representation matching ability [z = 2.06, *b* = 0.75, *p* < 0.05], which had a direct effect on emotion recognition accuracy [z = 6.93, *b* = 0.70, *p* < 0.001]. With respect to the latter, our analysis revealed that there were significant direct effects of (1) distance between emotion clusters on emotion recognition accuracy [z = 2.18, *b* = 0.20, *p* < 0.05], and (2) emotion recognition accuracy on distance between emotion clusters [z = 2.47, *b* = 0.24, *p* < 0.01], thus suggesting a bidirectional feedback loop between these variables. In addition, whilst distance between representations had a direct effect on distance between emotion clusters [z = 2.93, *b* = 0.28, *p* < 0.01], it did not exert an indirect effect on accuracy [z = 1.80, *b* = 0.05, *p* = 0.072]. Finally, our analysis also identified a significant direct effect of emotional consistency on non-verbal reasoning ability [z = 2.21, *b* = 0.63, *p* < 0.05], and of alexithymia on distance between clusters [z = − 2.27, *b* = − 0.15, p < 0.05] (Table 1).

**Table 1:** Parameter estimates for our final structural equation model.

Path	Estimate	z-value	*p*-value	Std. *b*
Representation matching → accuracy	0.021	6.932	< 0.001	0.700
Emotional consistency → representation matching	4.393	2.137	= 0.033	0.754
Emotional consistency → representation matching → accuracy *	0.090	2.052	= 0.040	0.527
Emotional consistency → NVR	0.018	2.205	= 0.027	0.633
Distance between clusters → accuracy	0.039	2.179	= 0.029	0.197
Accuracy → distance between clusters	1.193	2.466	= 0.014	0.236
Distance between representations → distance between clusters	2.611	2.932	= 0.003	0.275
Distance between representations → distance between clusters → accuracy *	0.102	1.800	= 0.072	0.054
TAS → distance between clusters	-0.058	-2.269	= 0.023	-0.150

Standardized betas are shown in the final column (Std. b). Indirect effects are labelled with an asterisk (*).

## Discussion

Here we illustrated that individual differences in the experience of emotion “on the inside” are interrelated with individual differences in representations of emotional expressions, and that these sources of individual differences predict 61% of the variance in emotion recognition accuracy. In Experiment 1 we developed (N = 20) and validated (N = 271) “EmoMap”, a novel method to map the shape of individuals’ emotional experience landscapes. In Experiment 2 we developed “ExpressionMap” to map the landscape of (N = 98; replication N = 193) participants’ representations of how emotions are expressed in the outside world. Subsequently we tested for a mapping between the experience of emotion “on the inside” and representations of the way emotions are expressed in the “outside world”. Individuals with modular internal emotion maps, who had consistent and distinct emotional experiences, tended to have consistent and distinct visual representations of other people’s dynamic emotional facial expressions. Structural Equation Modelling further illustrated that such individuals tended to have correspondingly enhanced emotion recognition accuracy. Therefore, our “*Inside Out Model of Emotion Recognition”* provides new insight into the psychological mechanisms underpinning individual differences in the recognition of emotion from dynamic facial expressions.

In our final model, which explained 60.8% of the variance in emotion recognition accuracy, there were two component processes that contributed to individual differences: the *consistency component* and the *differentiation component*. Within the consistency component, which explained a larger proportion of the variance in emotion recognition, those with less consistent emotional experiences also had less consistent visual emotion representations, and correspondingly low emotion recognition accuracy. Interestingly, representational consistency only contributed to emotion recognition for those with a lower ability to match two visually displayed expressions. With respect to the differentiation component, having poorly differentiated representations of others’ expressions, predicted poorly differentiated experiences of anger, happiness and sadness and corresponding difficulties with emotion recognition (note that this later link between experience and recognition of emotion was bi-directional). The directions of all the paths in our model were determined through systematic comparison of BIC scores. BIC comparisons revealed strong to very strong evidence that the directions in our final model were the most mathematically plausible. Nevertheless, it is important to note that structural equation modelling cannot definitively determine causality^35^. Thus, any directions of causality suggested by our model are merely hypotheses that should be tested via causal manipulation^36^.

Taking a step back from the individual path directions, it is pertinent to consider the *component processes* outlined in our final model. Although our modelling allowed for other pathways to emotion recognition difficulties—for example an emotion pathway (emotional consistency, distance between clusters) and a representation pathway (representational consistency, distance between representations)—our analyses demonstrated that the *consistency* and *differentiation* component processes were the most mathematically plausible. The emergence of these components is somewhat surprising given that EmoMap and Expression map were completed in two separate sittings (on two separate days) and that different methods were used to calculate their corresponding variables (see “Methods”). The emergence of these components *despite* their corresponding variables being calculated differently and measured across different sittings suggests that they are meaningful components of emotion recognition rather than methodological artefacts.

More generally, it is useful to consider alternative explanations for our conclusion that individual differences in emotion recognition from dynamic stimuli can, in part, be explained by individual differences in the way emotions are experienced and the way expressions are represented. A primary question concerns whether a third variable unrelated to emotion, such as participants’ motivation to do well, underpins the relationships between the experience, representation and recognition of emotion. In other words, do those with more consistent experiences of emotion also have more consistent visual emotion representations and more accurate emotion recognition simply because these individuals tried harder on all tasks? Our findings suggest that this is unlikely: self-reported effort was not significantly associated with emotional consistency, representational consistency, distance between representations, matching difficulty, or representation matching, respectively (all p > 0.05; see Supplementary Information C). In addition, although there were small-moderate correlations between effort and distance between clusters [R = 0.272, p < 0.001], and effort and emotion recognition accuracy [R = 0.236, p = 0.001] respectively, our Bonferroni-corrected partial correlations demonstrated that all the relationships we discovered remained significant after controlling for self-reported effort (see Supplementary Information C). Hence, the relationships we found between the experience, representation and recognition of emotion are not underpinned by self-reported effort. Similarly, since each of our paradigms included intricately designed attention checks, it is unlikely that differences in attention underpin the associations between these variables. Finally, one may ask whether the relationships documented here pertain specifically to the processing of emotion. That is, could it be that some individuals have consistent and distinct concepts in general and hence they are good at recognizing any complex stimuli. Our results suggest that this possibility is also unlikely: only those with consistent experiences of emotion, and not those with consistent concepts of color, had greater representational consistency and emotion recognition accuracy (see above and Supplementary Information B). Hence overall, it is unlikely that effort, attention, or another domain general process (e.g., having distinct concepts in general) underpin the associations found here. Rather our results, which have been acquired across several experiments with large samples (involving built-in replications), provide convincing evidence for links between the experience, representation and recognition of emotion.

But how do such links come about? For example, why would having inconsistent emotional experiences lead to inconsistent expression representations? As noted in our Introduction section, constructionist theories offer a theoretical framework that may help us to answer such questions. Constructionist theories of emotion (e.g.^15–20^) propose that children are continually constructing multimodal representations of emotions. For example, when hearing a caregiver describe a situation as “anger inducing” a child may associate their current internal sensations and prevailing visual/auditory/tactile inputs with the word “angry”. Over time “angry” ceases to be just a word and becomes a multimodal concept^15^ and once the concept is acquired, it may function to sharpen its own conceptual boundaries^14^. That is, having consistent and distinct emotion concepts may help a learner focus on invariant features of facial expressions and ignore between expression variation, thus encouraging the formation of consistent (i.e., reliable) and distinct visual representations of others’ expressions. Note that the reverse direction of causality is also possible: a child with consistent visual representations of others’ expressions may be better able to recognize when others are angry thus providing the child with a label with which to categorize their own internal states. Such a child may have more opportunities for labelling their internal states, potentially resulting in more consistent and distinct emotional experiences. As mentioned previously, further work must test the Inside Out Model’s directions of causality if we are to make more confident claims about the causal link between the experience, visual representation and recognition of emotion, and develop richer theoretical models of the developmental experiences that give rise to such links.

In addition to contributing to constructionist theories of emotion, our findings are also relevant to the face identity and signal detection literatures^7–9^. By demonstrating that consistent visual representations of emotional expressions facilitate the recognition of emotional expressions, we illustrate an important role for stored visual representations in *emotion* recognition, that extends beyond the known role of representations in facial *identity* recognition. Our findings are also partially in line with signal detection theory^10^. That is, we identified that emotion recognition was directly predicted by the consistency, but not the differentiation, of visual emotion representations. These findings raise the possibility that there are independent contributions of these factors to emotion recognition. The lack of a significant (direct) effect of the differentiation of visual emotion representations (i.e., the distance between attributed speed) on recognition may be due to the presence of large differences in the speeds attributed to angry, happy, and sad facial expressions [angry mean (SEM) = 3.85 (0.06); happy mean (SEM) = 2.80 (0.04); sad mean (SEM) = 1.63 (0.03)], meaning that on average the representations are ‘far apart’ and instances of overlap between the signal and noise distributions are relatively uncommon. Independent of this, there may feasibly be an additional effect of the consistency of visual emotion representations (variation in attributed speeds). For example, the expectation literature would predict that more precise (i.e., a representation that is consistent in appearance across instances) representations of upcoming stimuli would precipitate increased recognition accuracy (see^37–40^). Future research should aim to include other emotions (e.g., surprise, disgust, and fear), likely to populate other points on the speed continuum, to identify whether this illuminates an effect of the differentiation of visual emotion representations. In the current study, we were unable to include additional emotions due to testing constraints. Including surprise, disgust and fear would have increased the duration of our test battery to over eight hours (doubling the current testing time of four hours) and compromised our ability to test such large samples (due to limits on resources). We selected high and low arousal (anger/ happiness and sadness), and positively and negatively valenced (happiness and anger/sadness), emotions to cover different regions in arousal-valence space^21^.

### Implications

Our Inside Out Model raises a number of testable hypotheses that may help us better understand the etiology of the emotion recognition difficulties documented in numerous clinical conditions (e.g., depression, anxiety, psychosis, eating disorders, Parkinson’s disease, and autism spectrum disorder; see^41–47^). In the current study, we have illuminated two component processes that may contribute to these difficulties: the differentiation component and the consistency component. With respect to the former, differences in recognizing others’ emotions may be linked to difficulties differentiating one’s own emotional states; indeed preliminary evidence supports this pathway in the context of depression, anxiety, schizophrenia, anorexia nervosa and autism (as found in^25,48–52^). The consistency component, on the other hand, suggests the testable hypothesis that emotion recognition difficulties in clinical conditions linked to inconsistent emotional experiences—such as bipolar disorder and psychosis, which are associated with mood fluctuations^53,54^—may be mediated by the (in)consistency of visual representations of emotional expressions. Identifying mechanistic pathways that explain variation in emotion recognition may help us design tailored support systems with potential impacts upon psychosocial adjustment^55^ and psychological health and wellbeing^56^. Hence, future studies should aim to test these predictions.

### Limitations

The results of the current study are informative with respect to understanding the links between the experience, visual representation, and recognition of emotion from facial motion cues alone. Here, we have employed point-light displays, which provide a way of studying core dynamic cues (e.g., speed), while controlling other perceptual dimensions^57,58^, such as identity (e.g., gender, age, ethnicity, face attractiveness), depth, and pigmentation, which are all known to influence emotion recognition^59–61^. Although this allowed us to accurately assess the contribution of kinematic cues to visual emotion representations, and their subsequent effect on emotion recognition accuracy (without these other cues confounding the results), such tight control may limit the extent to which our findings generalize to full dynamic emotional expressions (e.g., full video recordings of facial expressions). It could be, for example, that the links we have demonstrated between the experience, representation and recognition of emotion exist for point-light displays, but not full emotional expressions. However, since individuals compare incoming facial expressions to stored templates, which represent the average facial expressions they have encountered previously (e.g., the average angry expression across all previous encounters^62–66^), it seems unlikely that the consistency of such templates would only be important when recognizing emotion from point-light displays (which are not typically encountered). Concurrently, there is no clear reason why an individual would draw on their own emotional experiences to recognize emotion, specifically in point light displays, and not in full dynamic expressions. Nevertheless, future studies are necessary to confirm whether our results generalize to full emotional expressions.

Relatedly, it is also worth noting that here we examine the consistency and differentiation of visual emotion representations specifically in the speed domain. This was an active design choice, motivated by previous evidence demonstrating the critical role of speed cues in the visual representation^67^ and recognition^22,32^ of emotion. Nevertheless, in future work, we will expand our paradigms to encompass other spatiotemporal emotion cues (e.g., degree of spatial exaggeration, movement onset/offset, texture, colour, etc.), thus facilitating investigations into the consistency and differentiation of visual emotion representations in other domains.

It is also important to consider the limitations of our EmoMap paradigm for assessing the experience of emotion. Although this paradigm has several methodological advantages—it can be completed online in just 25 to 35 minutes and does not require participants to translate their emotional experiences into words (see^68^)—there are disadvantages of using such computer-based assessments. For example, by employing images to elicit emotional reactions (as is common in the literature e.g.,^6,24,26,69,70^), participants may respond based on how they think they *should* feel, rather than how they truly feel. Whilst this is a possibility, we specifically address this issue in our task instructions, thus minimizing the likelihood of participants responding in this way: when describing EmoMap, we told participants that “this isn’t about what the image represents, or how you think other people, on average, respond to the images. It is about your own personal response”. Nevertheless, future investigations could benefit from employing more ecological methods such as experience sampling (e.g.,^71,72^), wherein participants label or rate their emotional state on several occasions throughout the day for multiple days. Using these methods, emotion differentiation can be calculated by computing intra-class correlations, measuring consistency between emotion ratings across occasions, for each participant (see^73^). Such studies could then aim to test whether the ability to differentiate emotions in everyday life is associated with more differentiated visual emotion representations, and enhanced emotion recognition.

Finally, it is important to highlight the limitations of our study with respect to sample generalizability. Across both experiments discussed here, the samples were predominantly female (74.91, 46.94, and 78.76% respectively), white (58.67, 83.67, and 56.48% respectively), and from the United Kingdom (37.27, 64.29, and 41.97% respectively). Since there may be differences in the experience and recognition of emotion between males and females^74–76^, it may be that the results discussed here are not representative of males. Although this is possible, the evidence from our post-hoc analyses suggest that our primary effects are not moderated by sex (see Supplementary Information D). Thus, it seems that for both males and females the experience, visual representation, and recognition of emotion are all linked. Nevertheless, further work should aim to verify our results in more balanced, and/or male, samples. In addition, previous studies have found that experiences (e.g.,^77–81^) and visual representations (e.g.,^82–85^) of emotion vary across cultures. Many of these studies suggest that there may be differences specifically in the *appearance* of visual representations (e.g., individuals from Western Cultures emphasize the eyebrows and mouth in their visual representations, while those from East Asian cultures the eye region^82^). Although this is an important consideration, it is worth noting that, in the current study, we specifically focus on the consistency and differentiation of visual representations, rather than on the appearance of them. Since individuals across numerous cultures employ template-matching techniques (i.e., comparing incoming facial expressions to stored ‘templates’) to recognise the emotions of others^62–66^, it seems unlikely that the *consistency* of such templates would be important in one culture but not another. Nevertheless, future studies should aim to the Inside Out Model across different cultures.

## Methods

### Experiment 1: developing EmoMap

This study was approved by the Science, Technology, Engineering and Mathematics (STEM) ethics committee at the University of Birmingham (ERN_16-0281AP9D) and was conducted in accordance with the principles of the revised Helsinki Declaration. Informed consent was obtained from all participants.

#### Participants

271 participants were recruited via the School of Psychology’s Research Participation Scheme database and Prolific. Individuals were eligible to take part in the study if they were between the ages of 18 and 65, fluent in English, had normal or corrected-to-normal vision, and had access to a computer/laptop with an internet connection. The sample size was based on an a priori power analysis conducted using G*power^86^. To replicate the association between alexithymia and emotion differentiation in Erbas et al.,^26^, 97 participants were required to have 95% power at alpha level 0.05. However, since effect sizes are commonly inflated^87^, and we were utilizing a more complicated analysis (a linear mixed effects model in which we control for the other relevant demographic variables known to be associated with the experience and perception of emotion), we recruited a larger number of participants (N = 271); almost triple the sample size generated in our power calculation).

Participant characteristics are displayed in Table 2. Information regarding participants’ ethnicities is reported in Supplementary Information E. Notably, four participants in the sample (1.48%) reported that they had a diagnosis of autism spectrum disorder. Therefore, we conducted our analyses twice, first including these participants and then excluding them. Since the general pattern of results was unaffected by their removal, we included these participants in our final statistical analyses.

**Table 2:** Means and standard deviations of participant characteristics.

Variable	Participants (N = 271)
Sex	68 male, 203 female
Age	24.00 (9.16)
NVR	60.22% (15.35%)
AQ-50	19.11 (6.85)
TAS-20	48.17(12.08)

In the column on the right-hand side, means are followed by standard deviation in parentheses.

#### Procedures

Participants completed demographics questions, followed by the Autism Quotient^88^ and Toronto Alexithymia Scale^89^ on Qualtrics (https://www.qualtrics.com). Subsequently, participants completed our EmoMap paradigm (openly available at https://app.gorilla.sc/openmaterials/447800) followed by the Matrix Reasoning Item Bank^90^ on Gorilla (https://gorilla.sc). All participants completed the study online.

#### Materials and stimuli

##### Demographics: questionnaires and tasks

The level of autistic traits of participants was assessed via the Autism Quotient^88^. The AQ is a 50-item questionnaire scored from 0 to 50, with higher scores representing higher levels of autistic characteristics. The level of alexithymic traits of participants was measured via the 20-item Toronto Alexithymia Scale^89^. The TAS comprises 20 items rated on a five-point Likert scale, ranging from 1, strongly disagree, to 5, strongly agree. Total scores on the scale range from 20 to 100, with higher scores indicating higher levels of alexithymic traits. Non-verbal reasoning ability was assessed via the Matrix Reasoning Item bank (MaRs-IB^90^). Each item in the MaRs-IB consists of a 3 × 3 matrix. In this matrix, eight of the nine available cells are filled with abstract shapes, and one cell in the bottom right-hand corner is left empty. Participants are required to complete the matrix by selecting the missing shape from four possible options. To correctly identify the answer, participants have to deduce relationships between the shapes in the matrix (which vary in shape, color, size and position). Non-verbal reasoning scores represent the percentage of trials in which participants provide the correct response.

##### EmoMap task

There were two key parts of the EmoMap task. In the first part, on each trial participants viewed pairs of images from the Nencki Affective Picture System^29^, and were instructed to “think about what feelings arise when you look at each of these images. Now please rate how SIMILAR those two feelings are”. Participants made their ratings on a visual analogue scale (with a step size of 0.0001) ranging from 0, ‘Not at all similar’ to 10, ‘Very similar’. An advantage of the EmoMap paradigm is that it provides a tool to measure emotion differentiation without requiring participants to produce emotion labels, unlike existing tasks (see^68,91^ for a full discussion).

The chosen images were known to be effective at selectively inducing anger, happiness or sadness across large samples (N = 124;^30^), and generated distinct emotion clusters based on graph theory analyses with pilot study data (see Supplementary Information F). In this task, there were five images for each emotion (anger, happiness and sadness) resulting in 15 different images and 105 unique image combinations (and therefore 105 trials): 30 within emotion-category combinations (10 for anger, 10 for happiness and 10 for sadness) and 75 between emotion-category combinations (25 angry-sad, 25 angry-happy, 25 happy-sad). A reaction time check was incorporated to prevent participants responding too quickly (i.e., without thinking). Responses faster than 1000 ms resulted in an error message (“Too Fast. Our algorithm has detected that you might need to take longer to think through your answer. You will now incur a 5 second penalty and then will be asked to do the trial again”) and a 5 second penalty, and then the trial was re-started. This threshold was selected to give participants sufficient time to view the images, detect and compare the emotions evoked by each of them, and then respond by clicking on the scale.

To map the shape and size of participants’ internal emotional landscapes, similarity ratings were transformed into (Euclidean) distance scores through multidimensional scaling (using the Scikit-learn library in Python). Multidimensional scaling (MDS) is a statistical technique that represents objects (emotional images, lexical items etc.) as points in multidimensional space wherein close similarity between objects corresponds to close distances between the corresponding points in the representation^92^. The distance between points in multidimensional space can then be plotted (see Fig. 1). Mean distances within specific emotion clusters comprised the average of the Euclidean distances for the 10 angry-angry, 10 happy-happy and 10 sad-sad image pairs, respectively. Mean distances between specific emotion clusters comprised the mean of the Euclidean distances for the 25 angry-happy, 25 angry-sad, and 25 happy-sad image pairs, respectively. We then computed mean distances within and between clusters by averaging across emotions/emotion pairs. Larger distances between and within emotion clusters reflect greater emotion differentiation.

The second part of our EmoMap paradigm was inspired by the work of Huggins and colleagues^93^. In this part of the task, on each trial, participants were presented with three images from the Nencki Affective Picture System^29^, and were required to make a decision. This task involved four conditions: one non-emotional control condition, and three emotional experimental conditions exploring the experience of anger, happiness and sadness respectively. First, participants completed the non-emotional control condition. In this condition, participants were required to select which of the three (neutral) images they found most colorful using their mouse cursor. Two of these images were in color and one was in grayscale, thus serving as an attention check. If participants selected the grayscale image, they were presented with an error message, incurred a 5 second penalty, and then were asked to do the trial again. Following this, participants completed the three experimental conditions in a random order. In these conditions, participants were required to select which of the three images made them feel most angry, happy or sad (e.g., in the angry condition, participants had to decide which of the two images made them most angry) using their mouse cursor. As was the case in the control condition, there was a ‘trap’ image on each trial in the emotional conditions. On each trial, two of the images were strong inducers of the target emotion (e.g., sadness), and one was a strong inducer of another emotion (e.g., happiness), thus serving as an attention check. If participants selected the image that strongly induced the non-target emotion, they were presented with the error message discussed above, incurred a 5second penalty, and then were asked to do the trial again. Within each condition, there were 11 target (i.e., non-trap) images which were presented in all possible unique pairs across 55 trials. The images that were selected had previously been identified as successful inducers of the target emotion^30^. In addition, in order to make the experimental conditions comparable, we ensured that the mean intensity ratings (angry = 3.53; happy = 3.50; sad = 3.56) and standard deviation of intensity ratings across images within a condition (angry = 0.80; happy = 0.80; sad = 0.81) were similar for each emotion.

Consistency scores were calculated for each condition in line with the logical consistency of a participants’ decisions. To illustrate, if a participant selects image A over image B (A > B) and image B over image C (B > C), these decisions are all consistent with one another. However, if the participant then selected image C over image A, this would be inconsistent with their previous judgments. Consistency requires participants to differentiate precisely between the intensity of emotion evoked by each image. Thus, inconsistent decisions are likely to stem from inconsistencies in how individuals experience an emotion across multiple instances.

We followed the procedures of Huggins et al.,^93^ to calculate consistency. We first quantified each participant’s image rankings by summing the number of times they chose each image. If a participant made completely consistent decisions in a set, rank scores would follow a linear sequence: the image they found most emotionally intense (or colorful) should be chosen in all ten trials it appeared (score = 10), the second-highest should be chosen in nine of ten trials (score = 9), and so on. The image they found least emotionally intense (or colorful) should never be chosen (score = 0). Subsequently, we examined how image rankings related to the decisions made on each trial. Since images with higher rank scores should elicit stronger emotional responses than those with lower rank scores, an inconsistent decision would occur when a lower-ranking image is chosen over a higher-ranking image. For each trial, the rank score of the unchosen item was subtracted from the rank score for the chosen item, producing item differences. For inconsistent decisions the item difference would be less than or equal to zero. More severe inconsistencies (e.g. choosing the lowest ranked image over the highest ranked image) result in more negative item differences. Item differences were then summed, per condition, to produce total consistency scores, with greater scores reflecting higher consistency. If a participant made no inconsistent decisions within a condition, their score would be 220.

### Experiment 2: developing ExpressionMap

#### Participants

The first (“original”) sample for Experiment 2 comprised 98 participants recruited via Prolific. The second, replication, sample comprised 193 participants recruited via the School of Psychology’s Research Participation Scheme database and Prolific. For both samples, individuals were eligible to take part if they were between the ages of 18 and 65, fluent in English, had normal or corrected-to-normal vision, and had access to a computer/laptop with Google Chrome and an internet connection. The sample size for our replication sample was based on an a priori power calculation using GLIMMPSE^94^. To have 90% power to replicate our finding from sample one that representational consistency predicted emotion recognition accuracy, 68 participants were necessary (alpha level = 0.05). Since effect sizes are commonly inflated^87^ and using larger samples improves the precision of parameter estimates^95^, we recruited a larger number of participants (N = 193; almost triple the sample size generated in our power calculation).

Participant characteristics are displayed in Table 3. Information regarding participants’ ethnicities is reported in Supplementary Information E. Notably, one participant in the original sample (1.02%) and two participants in the replication sample (1.02%) reported that they had a diagnosis of autism spectrum disorder. Therefore, we conducted our analyses both including, and then excluding, these participants. Since the general pattern of results was unaffected by their removal, we included these participants in our final statistical analyses.

**Table 3:** Means and standard deviations of participant characteristics.

Variable	Experiment 2, original sample (n = 98)	Replication sample (n = 193)
Sex	52 male, 46 female	41 male, 152 female
Age	33.34 (9.79)	23.41 (9.04)
NVR	58.45% (16.62%)	61.24% (14.79%)
AQ-50	18.65 (7.64)	18.94 (6.79)
TAS-20	46.00 (11.82)	48.13 (11.58)

In the column on the right-hand side, means are followed by standard deviation in parentheses.

#### Procedures

First, informed consent was obtained from all participants before conducting the study. Participants in the original sample completed demographics questions, followed by the 50-item Autism Quotient^88^, and the 20-item Toronto Alexithymia Scale^89^ on Qualtrics (https://www.qualtrics.com). Following this, these participants completed three tasks that employed dynamic point light displays (a series of dots that convey biological motion) of angry, happy and sad facial expressions (PLFs) on Gorilla (https://gorilla.sc). Participants completed ExpressionMap followed by the Visual Matching task, followed by the PLF Emotion Recognition task (openly available at https://app.gorilla.sc/openmaterials/447800). Finally, participants completed the MaRs-IB^90^. For those in the replication sample, participation was split across two parts. In part one, participants completed demographics questions, the AQ, TAS and EmoMap paradigm. In part two, which was completed in a separate sitting at least 24 hours after finishing part one, participants completed ExpressionMap, the Visual Matching Task, the PLF Emotion Recognition task and the MaRs-IB. For both samples, all parts of the study were completed online.

#### Materials and stimuli

##### ExpressionMap

In this task, on each trial, participants were presented with a dynamic point light display of the face (PLF; on average approximately 6 seconds in length) that looped such that it played continuously. Participants were instructed to “move the dial to change the speed of this video until it matches the speed of a typical ANGRY/HAPPY/SAD expression”.

The PLFs were originally created by asking actors to read a sentence (“my name is John and I’m a scientist”) in a happy, angry or sad manner^32^. The emotion depicted in the stimulus video matched the instructed emotion (i.e., on a trial where an angry facial expression was presented, participants were only asked to manipulate the speed of the video so that it matched the speed of a typical angry expression). Consequently, participants were matching the speed of the displayed PLF to their imagined visual representation of that expression (the speed they would imagine “in their mind’s eye”). Participants could change the speed of the video by moving a dial clockwise to increase the speed of the animation or anticlockwise to decrease the speed of the animation. The minimum and maximum point on the dial corresponded with 25% and 300% of the recorded speed respectively. Once participants were satisfied, they pressed the spacebar to continue. There was no time limit for participants to respond on each trial. Participants were shown four repetitions of each PLF stimulus video (each one starting at a random speed) across four actors. This resulted in 16 videos per emotion (4 actors × 4 repetitions × 3 emotions = 48 trial in total). Participants completed three practice trials (one for each emotion at 100% starting speed) and then the 48 randomly ordered experimental trials across three blocks. Participants were encouraged to take breaks between blocks.

The ExpressionMap task was adapted from Keating et al.^67^. In the current study we improved the task by (a) using a dial, instead of the slider used previously, thus making the minimum and the maximum points on the scale more ambiguous, (b) starting each video at a random speed thus reducing potential response biasing, (c) setting the initial dial position to a random orientation that did not correspond to starting speed, thus ensuring that the minimum and maximum points, and the point of the 100% recorded speed were at different spatial locations on the dial—as a result, participants were unable to be consistent simply by selecting a similar location on the dial each time—, (d) incorporating a reaction time check—when participants responded faster than 5 seconds on a trial, they were presented with an error message, incurred a 5 second penalty, and then were asked to do the trial again and, (e) incorporating a walk-through video to facilitate comprehension of task instructions.

Whereas existing methods aim to construct comprehensive representations of emotional expressions (e.g.^83,85,96^), ExpressionMap seeks to assess accompanying features of those representations (e.g., speed, consistency and differentiation; see^97^). ExpressionMap provides an index of the percentage speed attributed to each of the stimulus videos by participants (e.g., if a participant attributes 130% speed to an expression, their representation for that expression is 1.3 times faster than the recorded speed). Following the procedures outlined in Keating et al.^67^, we calculated the true speed attributed to each of the PLFs (in pixels per frame) by multiplying the percentage speed attributed, divided by 100, with the speed of the actor’s facial movement in the original video. For example, for a trial in which a participant attributed 200% speed to a face moving at 2.5 pixels/frame, the true speed attributed to the expression would be 5 pixels/frame [i.e., $\left(200\div 100\right) \times 2.5$] (see^67^ for more information).

This task operates on the premise that, compared to participants with consistent visual representations, those with less consistent representations of emotion would attribute more variable speeds to the expressions^98^. For instance, someone with a consistent visual representation of anger would attribute similar speeds across repetitions (e.g., by attributing 120% speed, 121% speed and 119% speed to the angry expression). In contrast, someone with a less consistent visual representation would be more variable (e.g., by attributing 120% speed, 60% speed and 180% speed to an angry expression). Therefore, to index the consistency of visual emotion representations, we took the standard deviation of the speeds attributed to one emotion for one actor (i.e., actor 1, angry expression) across the 4 video repetitions. Following this, we multiplied standard deviation scores by -1 so that our variable would now represent consistency (note that in Figs. 4 and 5 we also added a constant of 2.52, since the lowest score was -2.52, to facilitate interpretation). We then calculated mean representational consistency for each of the emotions (angry, happy and sad) by taking a mean of the consistency scores for each actor within an emotion (e.g., taking a mean of the consistency scores for angry expressions across actors 1, 2, 3 and 4). Mean representational consistency was calculated by taking a mean of the consistency scores for the angry, happy and sad PLFs.

Finally, this task also provides an index of the ‘distance’ between emotions in participants’ visual representations of facial expressions. To calculate distance scores, we subtracted the speed attributed to one emotion from the speed attributed to another and then took the absolute value of this number. For example, to calculate the distance between happy and angry PLFs, we subtracted the mean speed attributed to happy PLFs from the mean speed attributed angry PLFs, and then took the absolute value. Mean distance was calculating by taking a mean of the scores for the angry-happy, angry-sad, and happy-sad distances.

##### Visual matching task

We reasoned that an individual might have beautifully consistent mental representations of others’ expressions and still struggle to recognize others’ emotions due to an inability to match the incoming expression data with the stored representation. Thus, we developed the Visual Matching task to assess how well participants can visually match the speed of one expression to another (displayed) expression. Each trial began with a PLF stimulus video on the left-hand side of the screen. After this video had played once, the same PLF stimulus video also appeared on the right-hand side of screen, moving at a random speed, and continued to play in a loop. Participants were instructed to “move the dial to change the speed of the video on the right until it matches the speed of the video on the left”. Turning the dial clockwise increased speed. Turning the dial anticlockwise decreased speed. The minimum and maximum points on the dial corresponded with 25% speed and 300% of the recorded speed respectively (participants were not explicitly informed about this). Once the participant was satisfied, they pressed spacebar to continue. Participants were shown four repetitions of each PLF stimulus video for each of the four actors; each repetition had a different starting speed. In each full set of 16 (4 actors × 4 repetitions) stimulus videos for an emotion, the starting speed ranged from 50 to 200% of the recorded speed, in 10% increments (i.e., 50%, 60%, 70%, 80%, 90%, 100%, 110%, 120%, 130%, 140%, 150%, 160%, 170%, 180%, 190%, 200%). This range of starting speeds ensured that participants were able to match across a variety of speeds. Participants completed three practice trials (one for each emotion at 100% starting speed) and then the 48 randomly ordered experimental trials across three blocks. Participants were given the opportunity to take breaks between blocks.

The Visual Matching task provides an index of how well participants can visually match the speed of one expression to another. To calculate deviation scores, we subtracted the percentage speed attributed to the expression on the right from the percentage speed attributed to the expression on the left, and then took the absolute value.  As such, deviation scores provide a measure of how far away the speeds of the two animations were (irrespective of whether they attributed too high or too low speed). Finally, we calculated mean deviation scores by taking a mean of all of the absolute deviation scores. Higher deviation scores represent greater difficulties matching the two expressions.

##### PLF emotion recognition task

In the PLF Emotion Recognition task^32^, participants viewed dynamic PLFs, created from videos of four actors verbalizing sentences whilst posing three target emotions (angry, happy and sad). PLFs were adapted (see^32^ for further detail) to achieve three spatial movement levels, ranging from decreased to increased spatial movement (50%, 100% and 150% spatial movement), and three kinematic levels, ranging from reduced to increased speed (50%, 100% and 150% original stimulus speed). Each trial in this task began with the presentation of a (silent) PLF video displaying one of the three emotions, at one of the three spatial and three kinematic levels. After watching the video, participants were required to rate how angry, happy and sad the person was feeling on three visual analogue scales (presented in a random order) ranging from ‘Not at all angry/happy/sad’ to ‘Very angry/happy/sad’. Each trial lasted approximately 25 seconds. Participants completed three practice trials and then 108 randomly ordered experimental trials (12 per condition) across three blocks. Participants were encouraged to take a break between blocks.

The three emotion rating responses for each trial were transformed into magnitude scores from zero to ten (with zero representing a response of ‘Not at all’ and ten representing ‘Very’) to three decimal places. Emotion recognition accuracy scores were calculated by subtracting the mean of the two incorrect emotion ratings from the correct emotion rating. For example, for a trial in which a happy PLF was displayed, the mean rating of the two incorrect emotions (angry and sad) was subtracted from the rating for the correct emotion (happy). Mean emotion recognition accuracy was calculated by taking a mean of accuracy scores across all emotions and levels of the spatial and kinematic manipulation.

#### Transparency and openness

In this manuscript, we report how we determined our sample sizes, all data exclusions, all manipulations, and how we calculated all measures. All datafiles, data-processing code, analysis scripts, and tasks are openly available at https://osf.io/hd8u2/wiki/home/. The data were processed and analyzed using R (R Studio version 2021.09.2), Python (Jupyter Notebook version 6.4.8), and JASP (version 0.16). All our linear mixed effects models were conducted in R Studio using the lmer function (from the *lme4* package). For these models, we also used the Anova function (from the *car* package) to conduct a Type III ANOVA with a Kenward-Roger^99^ approximation for degrees of freedom, as supported by Luke^100^. For all linear mixed effects models, the relationships between the experience, representation, and recognition of emotion hold when the control variables are included (as reported in the Results section) and excluded, thus affording us greater confidence in our findings. In R Studio, we also conducted a random forest analysis^33^ employing the Boruta wrapper algorithm (Boruta function from *Boruta* package;^34^), and structural equation modelling using the sem() function (from the *lavaan* package). In JASP, we conducted Bayesian linear regressions to determine the relative strength of evidence for the experimental versus null hypotheses. For these analyses, we followed the classification scheme from Lee and Wagenmakers^101^: BF_10_ values between one and three reflect weak evidence, between three and ten moderate evidence, between ten and 100 strong evidence, and greater than 100 extreme evidence for the *experimental* hypothesis.

### Supplementary Information

Supplementary Information.

## Data Availability

All datafiles, data-processing code, analysis scripts, and tasks are openly available at https://osf.io/hd8u2/wiki/home/.
