# Supplementary material for: The inside out model of emotion recognition: how the shape of one’s internal emotional landscape influences the recognition of others’ emotions
Source: Sci Rep. 2023 Dec 6;13:21490. doi: 10.1038/s41598-023-48469-8 (PMC10700588; doi:10.1038/s41598-023-48469-8)
Supplement: Supplementary file 1 — Supplementary Information. [file 41598_2023_48469_MOESM1_ESM.docx]

**Supplementary Information**

**The Inside Out Model of Emotion Recognition: How the Shape of One’s Internal Emotional Landscape Influences the Recognition of Others’ Emotions**

**Authors**

Connor T. Keating^1^* & Jennifer L. Cook^1^

**Affiliations**

^1^School of Psychology, University of Birmingham, Birmingham, UK

**Supplementary Information A- The effect of emotion on emotional consistency and representational consistency, and the effect of emotion pair on distance between clusters and distance between representations.**

To assess whether the consistency of emotional experiences and visual emotion representations differed as a function of emotion, we constructed two linear mixed effects models. In the first model, emotional consistency was the outcome variable; in the second, representational consistency was the outcome variable. In both models, emotion (angry, happy, sad) was included as a predictor and subject number was modelled as a random intercept. Whilst representational consistency differed as a function of emotion [original sample: F(2,194) = 86.63, p < .001; replication sample: F(2,384) = 252.44, p < .001], emotional consistency did not [p > .05]. Across both samples, representational consistency was highest for sadness [original sample mean (SEM) = -0.51(0.03); replication sample mean(SEM) = -0.49(0.02)], followed by happiness [original sample mean(SEM) = -0.63(0.03); replication sample mean(SEM) = -0.62(0.02)], followed by anger [original sample mean(SEM) = -0.88(0.04), replication sample mean(SEM) = -0.88(0.03)].

Next, we aimed to assess whether distance between emotion clusters and distance between representations differed as a function of emotion pair. Therefore, we constructed two linear mixed effects models predicting distance between clusters, and distance between representations, respectively, with emotion pair (angry-happy, angry-sad, happy-sad), and with subject number as a random intercept. There was a significant main effect of emotion pair for distance between emotion clusters [F(2,540) = 487.69, p < .001]: there were smaller distances between anger and sadness [mean distance (SEM) = 14.39(0.21)], than happiness and anger [mean distance (SEM) = 20.79(0.29)], and happiness and sadness [mean distance (SEM) = 20.70(0.29)] in this experience domain. These results suggest that experiences of anger and sadness (i.e., same-valence emotions) are more similar than experiences of happiness and anger, and happiness and sadness (i.e., opposite-valence emotions).

****In addition, we found a significant main effect of emotion pair for distance between representations [F(2,384) = 180.44, p < .001]: there were smaller distances between representations for anger and happiness [mean(SEM) = 1.16(0.05) pixels/frame] and happiness and sadness [mean(SEM) = 1.18(0.04) pixels/frame], than for anger and sadness [mean(SEM) = 2.23(0.07) pixels/frame] in this speed domain. These results are logical: previous findings suggest that visual representations of anger are typically fastest, followed by happiness, followed by sadness (Keating, Sowden & Cook, 2022). Since happy expressions comprise an intermediate, they are most likely to overlap with both anger and sadness. To illustrate these effects, we computed a distance (i.e., dissimilarity) matrix for both EmoMap and ExpressionMap (see Figure S1). In this matrix, you can see that there are greater distances between experiences of anger and happiness, and smaller distances between experiences of anger and sadness in EmoMap. Conversely, there are smaller distances between visual representations for anger and happiness, and larger distances between representations for anger and sadness in ExpressionMap.

**Figure S1.** Two distance matrices illustrating the mean distances between emotional experiences for anger, happiness and sadness (for five images each; left) and the mean distances between visual representations of anger, happiness and sadness (for four repetitions each; right).

**Supplementary Information B- Building the Inside Out Model of Emotion Recognition**

***Determining which variables are important for emotion recognition***

To assess the contribution of both EmoMap and ExpressionMap variables to emotion recognition we focused on the 193 participants that completed both tasks. First, since we had a large number of potential variables of interest, we determined their relative importance with respect to emotion recognition using a random forest analysis (Breiman, 2001) employing the Boruta wrapper algorithm (Kursa & Rudnicki, 2010). Our random forests analysis (Breiman, 2001) employed the Boruta (Kursa & Rudnicki, 2010) wrapper algorithm (version 7.7.0) which trains a random forest regression model on all predictor variables, as well as their permuted copies (known as “shadow features”),and classifies a variable as important when its permutation importance is significantly higher than the highest permutation importance of a shadow feature. Our predictor variables included mean emotional consistency, color (control) consistency, mean distance between emotion clusters, mean distance within emotion clusters, mean representational consistency, matching difficulty, mean distance between emotion representations, and representation matching. ‘Representation matching’ was computed by multiplying the representational consistency scores for angry, happy and sad expressions with their corresponding matching difficulty scores (e.g., angry representational consistency x angry matching difficulty; happy representational consistency x happy matching difficulty; sad representational consistency x sad matching difficulty). Higher representation matching scores indicate superior representational consistency, matching ability, or both.

This analysis revealed that, of the eight variables, five were confirmed *important*, and three were confirmed *unimportant.* Figure S3 illustrates that representation matching [median importance score (MIS) = 21.58], matching difficulty [MIS = 21.40], representational consistency [MIS = 13.56], distance between emotion clusters [MIS = 7.46], and emotional consistency [MIS = 4.08] were classified as important (green) for emotion recognition. Mean distance between emotion representations [MIS = 1.36], mean distance within emotion clusters [MIS = -0.39], and colour control consistency [MIS = -1.46] were classified as unimportant (red).

******

**Figure S2*.*** Random forest variable importances*.* Variable importances of all eight features entered into the Boruta random forest, displayed as boxplots. Box edges denote the interquartile range (IQR) between first and third quartile; whiskers denote 1.5 * IQR distance from box edges; circles represent outliers outside of 1.5 * IQR above and below box edges. Box colour denotes decision: Green = confirmed, red = rejected; grey = meta-attributes shadowMin, shadowMax and shadowMean (minimum, maximum and mean variable importance attained by a shadow feature).

***Constructing the most mathematically plausible structural equation model***

Following this, we employed structural equation modelling (SEM) to build a mathematically plausible mechanistic model of the pathways linking internal emotional experiences with emotion recognition in the outside world. To achieve this, we first estimated latent constructs from their manifest indicator variables while accurately isolating any measurement error (Acock, 2013). The latent construct of emotional consistency was estimated using emotional consistency EmoMap scores for anger, happiness, and sadness respectively; distance between emotion clusters was estimated using the EmoMap distances between the angry and happy, angry and sad, and happy and sad clusters respectively; representation matching was estimated using ExpressionMap scores for the interaction between representational consistency and matching for angry expressions, representational consistency and matching for happy expressions, and finally representational consistency and matching for sad expressions; emotion recognition accuracy was estimated using accuracy scores from the PLF Emotion Recognition Task for angry, happy and sad expressions respectively. Due to failure of model convergence as a result of high collinearity between manifest variables [correlation for the distance between angry and happy, and angry and sad representations: R = .725, p < .005], the latent construct distance between representations was estimated using the distance between happy and sad, and angry and sad representations only (i.e., the distance angry and happy representations was not used to estimate distance between representations).

Subsequently, we modelled the structural (direct and indirect) paths between latent constructs. To this end, we added variables classified as “important” in our random forests analysis into a structural equation model predicting emotion recognition accuracy, sequentially (starting with the most important variable), until there was a) no longer a significant improvement (or our goodness of fit index exceeded the specified threshold), or b) our goodness of fit indices dropped below threshold (Root Mean Square Error of Approximation (RMSEA) > 0.08; Standardized Root Mean Square Residual (SRMR) > 0.08; Comparative Fit Index (CFI) < 0.95; Hu and Bentler, 1999). We also included paths for variables that were discovered to be significant predictors in our previous analyses (e.g., predicting emotional consistency with NVR, predicting distance between clusters with TAS). Within this model, there were significant direct effects of distance between emotion clusters [z = 3.96, *b* = 0.32, p < .001] and representation matching [z = 6.68, *b* = 0.69, p < .001] on emotion recognition accuracy. In addition, mediation analyses to test for the presence of indirect effects on accuracy revealed that emotional consistency contributed to accuracy [z = 2.11, *b* = 0.51, p < .05] by influencing the representational consistency x matching interaction [direct effect: z = 2.22, *b* = 0.73, p < .05]. Furthermore, serial mediation analyses identified that non-verbal reasoning exerted an indirect effect on accuracy [z = 4.99, *b*  = 0.33, p < .001] by influencing emotional consistency [direct effect: z = 2.24, *b* = 0.65, p < .05], which contributed to the representational x matching interaction [direct effect: z = 2.22, *b* = 0.73, p < .05], which predicted emotion recognition [direct effect: z = 6.70, *b* = 0.70, p < .001]. Finally, we identified that alexithymia exerted an indirect effect on emotion recognition accuracy [z = -2.23, *b* = -0.06, p <.05] by influencing distance between emotion clusters [z = -2.68, *b* = -0.19, p < .01], which in turn contributed to distance between emotion representations [direct effect: z = 2.60, *b* = 0.26, p < .01].

A significant strength of structural equation modelling is that it provides the opportunity to reverse path directions to establish mathematically plausible directions of causality (Kline, 2015). We constructed a series of structural equation models in which the direction of one (and only one) of the paths was reversed and calculated Bayesian Information Criterion (BIC) difference scores by subtracting the sample size adjusted BIC scores of our final model from the ‘reversed models’. BIC difference scores between 2 and 6 reflect moderate evidence, between 6 and 10 reflect strong evidence, and above 10 reflect very strong evidence, for model improvement (Raftery, 1995). There was *very strong* evidence that our model was better than the reversed model in three instances (representation matching → emotional consistency: BIC difference = 50.953; accuracy → representation matching: BIC difference = 16.219; distance between clusters → TAS: BIC difference = 1496.51). However, interestingly, there was very strong evidence that the reverse direction was more plausible in one instance (emotional consistency → non-verbal reasoning: BIC difference = -184.898). Finally, our model and the reversed model were comparable in two instances (emotion recognition accuracy → distance between clusters: BIC difference = 0.953; distance between emotion representations → distance between clusters: -1.841; see Table S2). Following this, we constructed a structural equation model in which we included the path directions that were mathematically most plausible (i.e., reversed one of the paths such that it was emotional consistency → non-verbal reasoning). For paths in which neither direction was more plausible, we modelled both path directions (i.e., distance between clusters → emotion recognition accuracy, and emotion recognition accuracy → distance between clusters; distance between emotion clusters → distance between emotion representations, and distance between emotion representations → distance between emotion clusters) in direct feedback loops.

**Table S1**. A table showing the difference in Bayesian Information Criterion (BIC) scores between our final structural equation model and models in which each of the paths were reversed.

| **Reversed Path** | **BIC Difference** | **Preferred Model** | **Strength of evidence** |
| --- | --- | --- | --- |
| Distance between clusters → TAS | 1496.51 | Original | Very strong |
| Representational Consistency x Matching → Emotional Consistency | 50.953 | Original | Very strong |
| Accuracy → Representational Consistency x Matching | 16.219 | Original | Very strong |
| Accuracy → Distance between clusters | 0.953 | Neither | No evidence |
| Distance between representations → Distance between clusters | -1.841 | Neither | No evidence |
| Emotional Consistency → NVR | -184.898 | Reversed | Very strong |

Notably, this model revealed that only one of these bidirectional feedback loops were significant: there were significant direct effects of distance between emotion clusters on accuracy [z = 2.26, *b* = 0.20, p < .05], and accuracy on distance between emotion clusters [z = 2.65, *b* = 0.27, p < .01], thus confirming a bidirectional feedback loop between these variables. By contrast, there was a marginally significant direct effect of distance between representations on distance between emotion clusters [z = 1.75, *b* = 0.54, p = .081], but not distance between emotion clusters on distance between representations [p = .396]. Therefore, we constructed one final structural equation model with the most mathematically plausible path directions, including a bidirectional feedback loop between distance between emotion clusters and accuracy, and a unidirectional path from distance between representations to distance between emotion clusters. There was very strong evidence that our final model, which could account for 60.8% of the variance in emotion recognition accuracy, was more mathematically plausible than our original model (BIC difference = 192.427). The information about our final structural equation model is reported in the Results section.

**Supplementary Information C- Partial correlations controlling for self-reported effort**

At the end of the study, we asked participants to report how much effort they put in while completing the tasks on a scale from 0 (no effort at all) to 10 (maximum effort). In order to elicit honest responses, we informed participants that they would still be renumerated for their time irrespective of their answer, and emphasized the importance of giving truthful responses.

In order to assess whether our variables of interest were associated with self-reported effort, we ran a series of simple correlations (see Table S3). This revealed that self-reported effort was not associated with emotional consistency, representational consistency, distance between representations, matching deviation scores, or representation matching [all p > .05]. However, there were small-moderate correlations between self-reported effort and distance between clusters [R = .263, p < .001] and emotion recognition accuracy [R = .236, p = .001].

**Table S2**. A table showing the Pearson correlations between self-reported effort and our variables of interest. Note that these p values are not corrected for multiple comparisons.

|  | Emotional consistency | Distance between clusters | Representational consistency | Distance between representations | Matching deviation | Representation matching | Emotion recognition accuracy |
| --- | --- | --- | --- | --- | --- | --- | --- |
| Effort | R = -.065  p = .368 | R = .272*  p < .001 | R = .071  p = .330 | R = .035  p = .631 | R = -.126  p = .081 | R = .110  p = .129 | R = .236*  p = .001 |

Therefore, in order to determine whether self-reported effort underpinned the relationships between our variables of interest, we conducted a series of partial correlations controlling for self-reported effort. Across all analyses, significant relationships were identified even after controlling for self-reported effort: the relationship between emotion recognition accuracy and distance between clusters [R = .260, *p_bon_*_f_ = .002], emotion recognition accuracy and the representational consistency x matching interaction [R = .549, *p_bonf_* < .001], the representational consistency x matching interaction and emotional consistency [R = .251, *p_bonf_* = .003], emotional consistency and non-verbal reasoning [R = .228, *p_bonf_* = .009], distance between representations and distance between clusters [R = .221, *p_bonf_* = .013], and distance between clusters and alexithymia [R = -.199, p*_bonf_* = .036], all held after Bonferroni-correction (correcting for six tests).

**Supplementary Information D – The effect of sex on our pattern of results**

Since our samples were unbalanced with regards to sex, we conducted a series of analyses to determine whether sex moderated any of our primary effects. The general pattern of results was very similar to that reported in the main manuscript (see full results below).

First, we conducted analyses assessing the extent to which the contribution of alexithymia to distance between and within clusters was moderated by sex. To test this, we constructed two linear mixed effects models with distance between clusters and distance within clusters as the outcome variables, TAS, sex, and the TAS x sex interaction as predictors, and with subject number as a random intercept. For distance *between* clusters, there was a significant effect of TAS [F(1,267) = -5.24, p < .05] that was not moderated by sex [p = .231]. As found previously, those higher in alexithymia had smaller distances between their emotion clusters. For distance *within* clusters, there was a significant TAS x sex interaction [F(1,267) = -4.80, p < .05]. Unpacking this interaction revealed that alexithymia was a significant negative predictor of distance within clusters for males [F(1,66) = 5.20, p <.05] but not females [p = .484]. It is important to note that this finding does not significantly change our main pattern of results; for our final structural equation model, TAS is modelled as a significant predictor of distance *between* clusters, while distance within clusters is not included in the model.

Next, we aimed to assess whether sex moderated the effect we found of distance between clusters, and distance within clusters on emotional consistency. Therefore, we constructed a linear mixed model predicting emotional consistency with distance between clusters, distance within clusters, their interactions with sex, and sex. As reported in the main manuscript, distance between clusters was a significant positive predictor [F(1,265) = 8.45, p <.01],and distance within clusters was a significant negative predictor [F(1,265) = -9.84, p<.01] of emotional consistency. There were no significant interactions with sex [p > .05], thus suggesting that these predictive relationships exist for both males and females.

Following this, we aimed to verify whether sex moderated the effect of representational consistency on emotion recognition accuracy. Thus, we constructed a linear mixed effects model of emotion recognition accuracy as a function of mean representational consistency, sex, and the representational consistency x sex interaction. Across both samples, there was a significant effect of representational consistency on emotion recognition accuracy [original sample: F(1,94) = 5.07, p < .05; replication sample: F(1,189) = 42.95, p < .001], that was not moderated by sex [all p > .05]. These results suggest that, for both males and females, representational consistency significantly contributes to emotion recognition accuracy.

Next, we aimed to assess whether sex moderated the effect of the representational consistency x matching interaction on emotion recognition accuracy. To fulfil this aim, we conducted a linear mixed effects model predicting emotion recognition accuracy with representational consistency, matching difficulty, the representational consistency x matching interaction, and the interactions of these variables with sex. In line with the results reported in the main manuscript, this identified a significant representational consistency x matching interaction [F(1,185) = 12.19, p < .001], that was not moderated by sex [p > .05]. Unpacking this interaction, revealed that representational consistency was only a significant predictor for those with a lower ability to match [F(1,92) = 17.53, p < .001], and not those with a higher ability to match [p > .05]. Again, these effects were not moderated by sex. Together, these results suggest that, for both males and females, when individuals struggle to match two expressions, representational consistency plays an important role.

Following this, we aimed to determine whether the relationships we discovered between how individuals feel “on the inside” and how they expect expressions to look “on the outside” were moderated by sex. First, we assessed whether the contribution of emotional consistency to representational consistency was moderated by sex using a linear mixed effects model. As found previously, emotional consistency was a significant predictor of representational consistency [F(1,189) = 12.46, p < .001]. There was no emotional consistency x sex interaction [p > .05], thus indicating that for both males and females, having consistent emotional experiences is associated with consistent visual representations of emotion. Second, we examined whether the contribution of distance between clusters to distance between representations was moderated by sex using a linear mixed model. This identified that, distance between clusters was a significant predictor of distance between representations [F(<1189) = 4.97, p < .05]. Importantly, this effect was not moderated by sex [p > .05]. Thus, for both males and females, having more distinct experiences of emotion predicts more distinct visual representations of emotion.

Next, we aimed to confirm whether the paths stipulated in our final structural equation model were present for both males and females. Therefore, we attempted to conduct independent structural equation models in each group. However, due to the sample of males being relatively small (N = 41), the model conducted in this sample did not converge. Therefore, it was not possible to assess the relationships between our variables of interest simultaneously in one model (for males). Nevertheless, it is reassuring that the evidence from our previous models points towards a consistency component that is not moderated by sex: we found significant relationships between emotional consistency and representational consistency, representational consistency and accuracy, and the representational x matching interaction and accuracy, independent of sex. We have also identified some evidence for the differentiation component existing for both male and females: we found significant relationships between alexithymia and distance between clusters, and distance between clusters and distance between representations, that are not moderated by sex. Further research, which employs larger samples of males, is necessary to determine whether the same mechanisms are involved in emotion recognition for both males and females.

**Supplementary Information E – Participants’ ethnicity information**

**Table S3**. A table displaying the ethnicities of participants from Experiment 1.

| **Ethnicity** | **Frequency** |
| --- | --- |
| Afghan | 1 |
| Arab | 2 |
| Asian Bangladeshi | 2 |
| Asian British | 11 |
| Asian Filipino | 1 |
| Asian HongKonger | 1 |
| Asian Indian | 8 |
| Asian Indonesian | 1 |
| Asian Nepali | 1 |
| Asian Pakistani | 10 |
| Asian Sri Lankan | 1 |
| Asian British Pakistani | 1 |
| Black African | 37 |
| Black African and Caribbean | 1 |
| Black British | 3 |
| Black Caribbean | 1 |
| Black/African/Caribbean background: Somali | 1 |
| Chinese | 18 |
| Cypriot | 1 |
| Hispanic | 1 |
| Mixed/Multiple ethnic groups- Asian/African | 1 |
| Mixed/Multiple ethnic groups- Indian/Bangladeshi/Iraqi | 1 |
| Mixed/Multiple ethnic groups- Middle East/Israeli | 1 |
| Mixed/Multiple ethnic groups- Portuguese/Arab | 1 |
| Mixed/Multiple ethnic groups- Latin American | 1 |
| Mixed/Multiple ethnic groups- White and Asian | 2 |
| Mixed/Multiple ethnic groups- White and Black African | 2 |
| White Albanian | 1 |
| White American | 2 |
| White Austrian | 1 |
| White Baltic Finnic | 1 |
| White Belgian | 1 |
| White Bulgarian | 1 |
| White Caucasian | 1 |
| White Czech | 1 |
| White Dutch | 1 |
| White Eastern European | 2 |
| White English/Welsh/Scottish/Northern Irish/British | 101 |
| White English/White Eastern European | 1 |
| White Estonian | 1 |
| White European | 5 |
| White French | 1 |
| White German | 1 |
| White Hispanic | 2 |
| White Iberian | 1 |
| White Irish | 1 |
| White Italian | 1 |
| White Latin | 1 |
| White Latvian | 1 |
| White Mediterranean | 1 |
| White Northern European | 1 |
| White Polish | 3 |
| White Portuguese | 5 |
| White Romanian | 2 |
| White Slaav | 3 |
| White South African | 2 |
| White Turkish | 1 |
| Not disclosed | 12 |
| **Total** | **271** |

**Table S4**. A table displaying the ethnicities of participants from Experiment 2, Original Sample.

| **Ethnicity** | **Frequency** |
| --- | --- |
| Asian Bangladeshi | 1 |
| Asian Indian | 3 |
| Asian Korean | 1 |
| Asian Pakistani | 1 |
| Black African | 3 |
| Black British | 1 |
| Black Caribbean | 1 |
| Chinese | 1 |
| Mixed/Multiple ethnic groups- White and Asian | 2 |
| Mixed/Multiple ethnic groups- White and Black Caribbean | 1 |
| White Caucasian | 1 |
| White English/Welsh/Scottish/Northern Irish/British | 63 |
| White European | 5 |
| White German | 1 |
| White Irish | 4 |
| White Italian | 1 |
| White Lithuanian | 1 |
| White Mixed European | 1 |
| White Polish | 2 |
| White Portuguese | 3 |
| Not disclosed | 1 |
| **Total** | **98** |

**Table S5**. A table displaying the ethnicities of participants from Experiment 2, Replication Sample.

| **Ethnicity** | **Frequency** |
| --- | --- |
| Afghan | 1 |
| Arab | 1 |
| Asian Bangladeshi | 2 |
| Asian British | 7 |
| Asian HongKonger | 1 |
| Asian Indian | 6 |
| Asian Indonesian | 1 |
| Asian Nepali | 1 |
| Asian Pakistani | 6 |
| Asian Sri Lankan | 1 |
| Asian: British Pakistani | 1 |
| Black African | 24 |
| Black African and Caribbean | 1 |
| Black British | 3 |
| Black Caribbean | 1 |
| Black/African/Caribbean background: Somali | 1 |
| Chinese | 12 |
| Cypriot | 1 |
| Mixed/Multiple ethnic groups- Asian/African | 1 |
| Mixed/Multiple ethnic groups- Indian/Bangladeshi/Iraqi | 1 |
| Mixed/Multiple ethnic groups- Portuguese/Arab | 1 |
| Mixed/Multiple ethnic groups- Latin American | 1 |
| Mixed/Multiple ethnic groups- White and Asian | 1 |
| Mixed/Multiple ethnic groups- White and Black African | 2 |
| White Albanian | 1 |
| White American | 1 |
| White Baltic Finnic | 1 |
| White Belgian | 1 |
| White Bulgarian | 1 |
| White Caucasian | 1 |
| White Czech | 1 |
| White English/Welsh/Scottish/Northern Irish/British | 81 |
| White Estonian | 1 |
| White European | 3 |
| White French | 1 |
| White Hispanic | 1 |
| White Irish | 1 |
| White Italian | 1 |
| White Latvian | 1 |
| White Northern European | 1 |
| White Polish | 3 |
| White Portuguese | 3 |
| White Romanian | 2 |
| White Slaav | 2 |
| White South African | 1 |
| Not disclosed | 6 |
| **Total** | **193** |

**Supplementary Information F - Pilot Study (N = 20)**

In the second part of the EmoMap paradigm, which assesses emotional consistency, there are 11 images that induce anger, happiness and sadness respectively (33 images in total). If we were to include all of these images in the first part of the EmoMap task, which assesses emotion differentiation, participants would be required to complete 528 trials (one trial for every image pair combination). Given that providing a similarity rating for each image pair combination typically takes 15 seconds, the duration of this task would be over two hours. Since this task is part of a wider battery investigating the experience, visualization and recognition of emotion, it is crucial that the task is shorter in length. Therefore, in a pilot study we aimed to identify five images for each emotion (15 images in total) that were effective at inducing the target emotion, and generated well-differentiated emotion clusters. By selecting five images per emotion, we knew that there would be 105 image pair combinations and therefore the task would take approximately 25 minutes to complete.

To fulfil our aim, we recruited 20 participants from Prolific (participant demographics shown in Table S1) to complete a longer version of the first part of the EmoMap task that included all 11 possible images for each emotion. In this task, on each trial participants viewed pairs of emotional images and were required to rate how similar the emotions evoked by the images were (see full task description in the main manuscript). To map the shape and size of participants’ internal emotional landscapes, similarity ratings were transformed into Euclidean distance scores through multidimensional scaling (using the Scikit-learn library in Python). These distance scores were then used to plot the internal emotional landscape (see Figure S1.). After completing the first part of the EmoMap task, participants also completed the ‘Emotion Label’ task. In this task, on each trial participants viewed one of the images they had seen previously and were then required to state the emotion they felt most strongly when looking at this image.

**Table S6.** Means and standard deviations of participant characteristics. In the column on the right-hand side, means are followed by standard deviation in parentheses.

| Variable | Participants (N = 20) |
| --- | --- |
| Sex | 9 Male, 11 Female |
| Age | 32.35(11.74) |
| AQ-50 | 22.40(7.23) |
| TAS-20 | 50.50(16.04) |


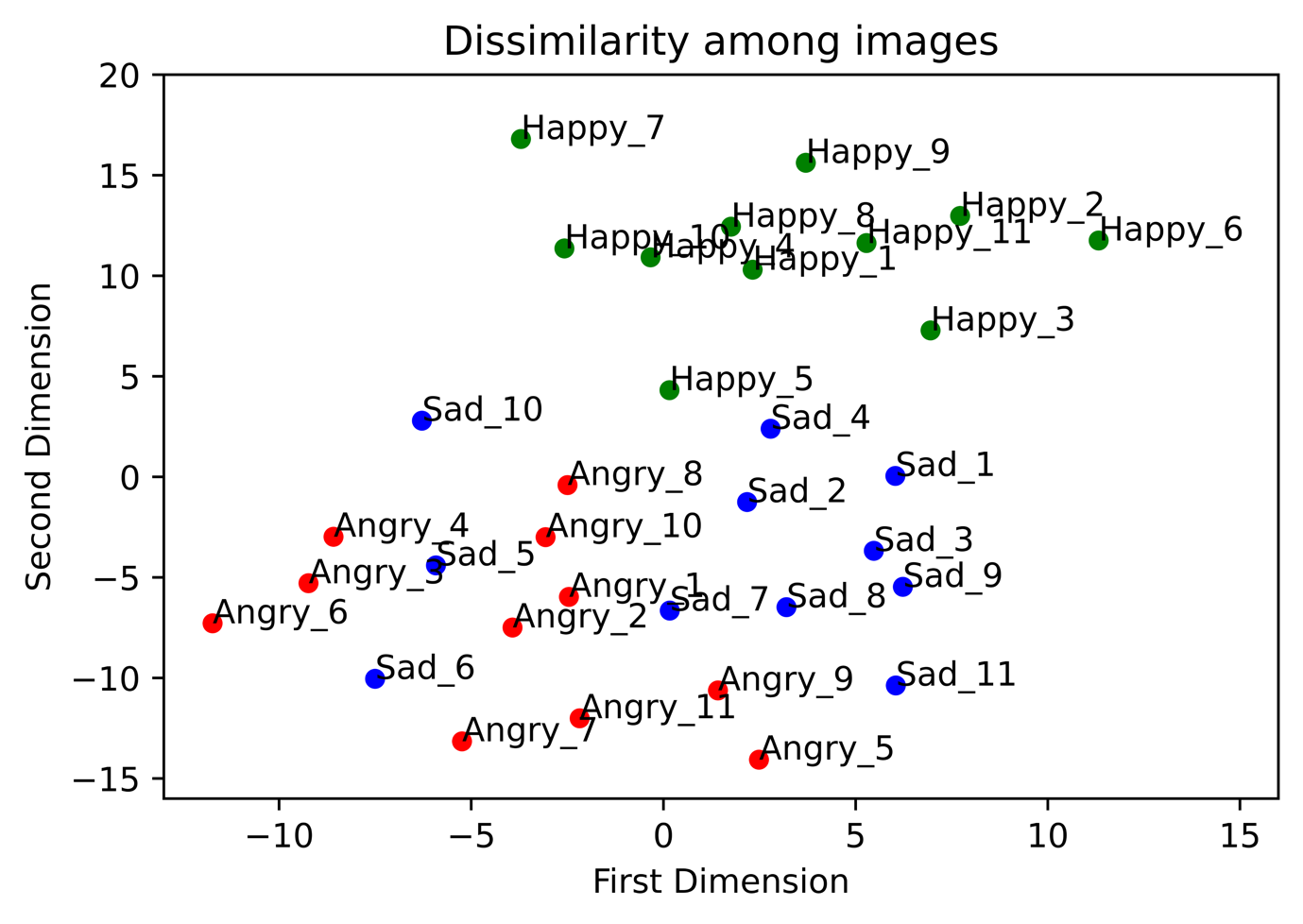


**Figure S3*.*** A diagram displaying the aggregated internal emotional landscape across all pilot study participants.

Following creation of the multidimensional scaling plot, we selected five images for each emotion based on the following criteria. Firstly, all of the selected images were rated as inducing the target emotion more than any other emotion in Riegel et al., (2016; e.g., for images selected to induce anger, the intensity rating for anger was higher than for all other emotions). Secondly, the selected images formed an emotion cluster that was visually distinct from the other clusters (this led to us exclude Sad_5, and Sad_6 as these sad images were close to many of the angry images in the map, and received relatively high angry ratings in Riegel et al., 2016). Thirdly, the images were freely labelled as inducing the target emotion (or a similar emotion, i.e., anger, frustration) by a higher number of participants than for unselected images on our independent emotion labelling task. Finally, we ensured that there was a similar mean intensity rating and standard deviation of intensity ratings for each emotion cluster based on the ratings from Riegel et al., (2016) [angry mean(SD) = 4.05(0.73); happy mean(SD) = 4.00(0.78); sad mean(SD) = 3.75(0.67)]. By doing so, it would not be the case that, for instance, there were larger distances within one emotion over another because there was a large difference in intensity ratings (and therefore the experience of emotion was less similar). The selected images for each cluster are shown in color (see Figure S2).


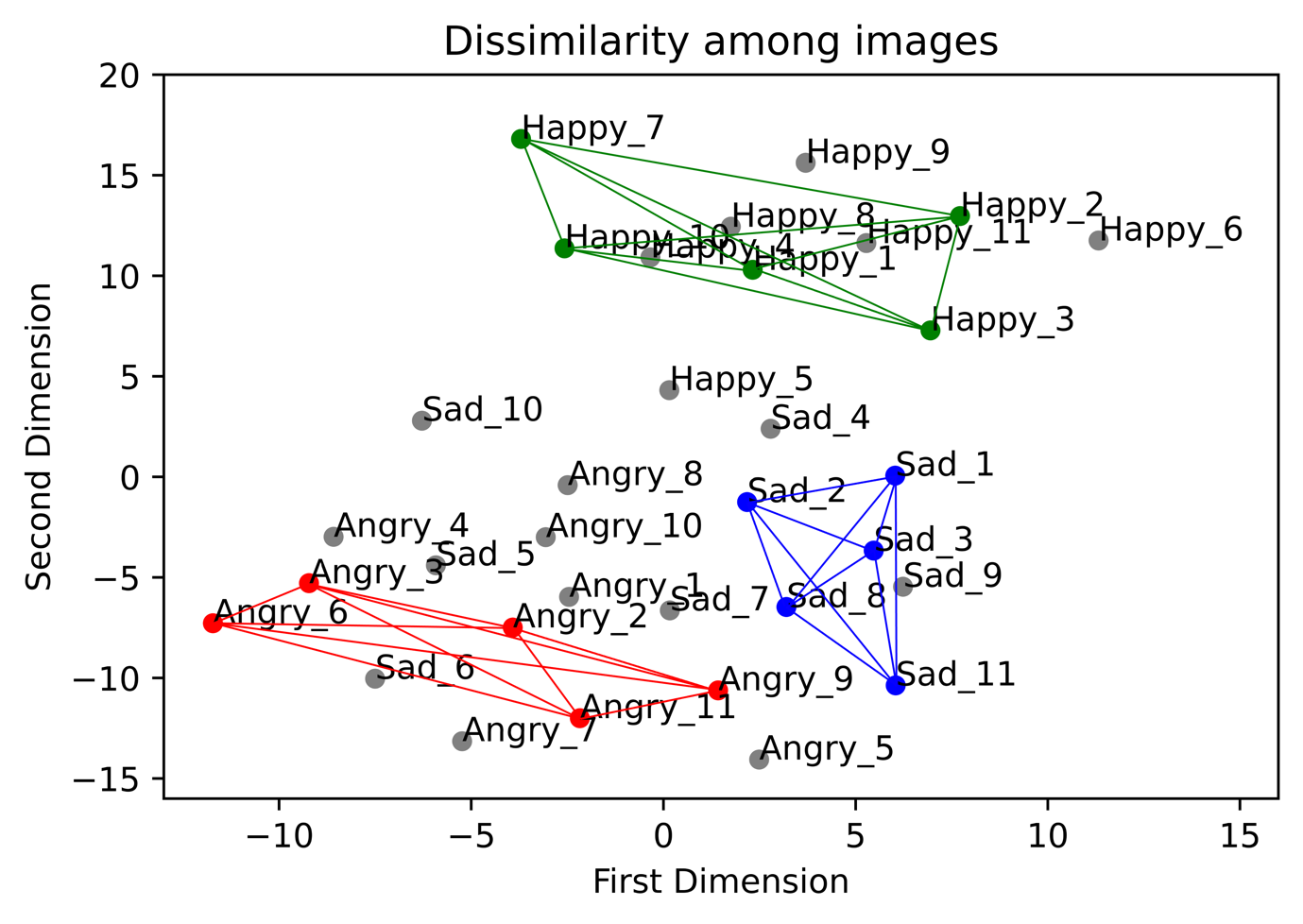


**Figure S4.** A diagram displaying the aggregated internal emotional landscape of pilot study participants. The images that were selected for the short version of the similarity task are in colour, and the images that were not selected are in grey.
